# Mutation-induced structural and dynamic perturbations at the IL-17A–IL-17RA interface: Therapeutic insights from multi-replicate molecular dynamics simulations

**DOI:** 10.1371/journal.pone.0361876

**Published:** 2026-11-06

**Authors:** Afsheen Razzaq, Madiha Sardar, Mamona Mushtaq, Mohammad Nur-e-Alam, Muhammad Nabeel Ghayur, Zaheer Ul-Haq

**Affiliations:** 1 H. E. J. Research Institute of Chemistry, International Center for Chemical and Biological, Sciences, University of Karachi, Karachi, Pakistan; 2 Dr. Panjwani Center for Molecular Medicine and Drug Research, International Center for Chemical and Biological Sciences, University of Karachi, Karachi, Pakistan; 3 Department of Pharmacognosy, College of Pharmacy, King Saud University, Riyadh, Saudi Arabia; 4 Kentucky College of Osteopathic Medicine, University of Pikeville, Pikeville, Kentucky, United States of America; Indian Institute of Management Bangalore, INDIA

## Abstract

The interaction between Interleukin-17A (IL-17A) and its receptor IL-17RA plays a key role in pro-inflammatory signal transduction and is implicated in the pathogenesis of inflammatory autoimmune diseases. Blocking this protein-protein interaction is a major approach to developing therapeutic agents to inhibit IL-17A-induced signaling. Predicting the consequences of mutations on structure and function in proteins can be accomplished by using computational methods, which are relatively inexpensive and rapid compared with experimental methods. Here, we used a multidimensional in silico approach to gain insight into the functional and thermodynamic consequences of targeted mutation of hotspot residues of IL-17A. The focus of our analysis was on the residues in the two chains that are equivalent to hotspot residues of the related cytokine, IL-17F, at positions 55 and 67 of chain B and 85 and 86 of chain A. Comprehensive analysis of the conformational dynamics, interaction profiles, and structural transitions of the four mutant variant systems (R55V, W67V, Y85I, and H86S) and the wild-type system was performed based on molecular dynamics (MD) simulations and Molecular Mechanics/Generalized Born Surface Area (MM/GBSA) calculations. A total of 3 μs simulations were performed: three 200 ns replicates for each of the wild-type and mutant complexes. Results from principal component analysis (PCA) and free energy landscape (FEL) analyses indicate conformational destabilization of the mutant systems. These findings help to elucidate the mechanisms of molecular interactions involved in the dynamics of cytokine–receptor interaction. Changes in these important PSN network parameters resulted in differences in hub connectivity and distribution of the links between the systems, which suggests allosteric perturbations and remodeling of the system in the interaction between IL-17A and IL-17RA when compared to the wild-type systems. The multiple-replica MD simulation protocol provided insight into how mutations affected the structure, contact networks, dynamics, and binding affinity of the IL-17A/IL-17RA complex, shedding light on the IL-17A downstream signaling cascades. Overall, this study provides a fundamental framework for designing therapeutic strategies to regulate IL-17A-mediated inflammatory diseases.

## 1. Introduction

Macromolecular interactions are involved in a wide range of biological activities, including signal transduction, proliferation, and DNA repair [1]. They are also implicated in a wide range of human diseases and represent attractive targets for therapeutic intervention to produce lead compounds. Their complex nature, due to the flat, relatively smooth topography and discontinuous nature of protein-protein interactions (PPIs) interfaces and structural flexibility, poses a great challenge for conventional structure-based drug design [2–4]. Despite this challenge, advances in technology over the last two decades have made “undruggable” targets more accessible. But the design of effective macromolecular interaction modulators is still a challenge. An effective strategy should include a combination of rational drug design, co-crystal structures, and computational methods. Mapping binding hot-spots through mutational studies can give insights into structural and kinetic contributions of contact residues, and can be used to target the pathways of disease and modulate PPIs [5–8].

A potential macromolecular interaction of particular interest is the cytokine Interleukin-17A (IL-17A), a key member of the IL-17 cytokine family, comprising IL-17A to IL-17F [9–13]. IL-17A was originally found in 1993 in an activated T cell hybridoma [14,15], and it is a key player in autoimmune inflammatory processes [16–18]. IL-17A is mainly produced by TH17 cells (CD4 + T-helper) and shares significant sequence similarity with other IL-17 cytokines, especially in the cytokine family’s C-terminal region, which displays conserved cysteine residues and 20−30% sequence identity [19,20]. This 150-amino acid cytokine is encoded on chromosome 6p12 and, besides TH17 cells, is also produced by CD8 + cytotoxic T cells (Tc17), natural killer (NK) cells, and group 3 innate lymphoid cells (ILC3s) [21–23]. The IL-17A/IL-17RA crystal structure demonstrates a large, relatively flat binding surface that comprises multiple contact patches as an example of its unique topography [24]. This topography restricts the development of pockets, preventing the design of small molecules that can achieve high-affinity and selectivity [25].

IL-17A contributes to autoimmune diseases through activation of the neutrophils and macrophages, induction of pro-inflammatory cytokines, and tissue damage. In cancer, it boosts anti-tumor responses by activating cytotoxic T cells and natural killer (NK) cells, but also establishes an immunosuppressive tumor microenvironment that facilitates malignant growth. It promotes the recruitment of Myeloid-Derived Suppressor Cells (MDSCs) to tumors, induces IL-6 secretion, and activates STAT3 signaling. IL-17A exists as homodimers and heterodimers with IL-17F and binds to the receptor IL-17RA via a large polar interface. The receptor-ligand recognition is crucial in the immune response to fungal and bacterial pathogens, and in autoimmune diseases and cancer development [19,22,24,26–29]. Specifically, IL-17A has been implicated in various disease conditions such as ankylosing spondylitis, psoriatic arthritis and psoriasis, and therapeutic approaches to inhibit the IL-17A/IL-17RA interaction have been successful [30]. Antibodies, such as Secukinumab and Ixekizumab, which target IL-17A, have been developed for these diseases [31–33]. Despite these improvements, there are still challenges in the clinical use of IL-17A inhibitors to achieve the best therapeutic outcomes.

IL-17A inhibitors have shown efficacy in inflammatory diseases, but have limitations of non-responsiveness, resistance, and limited long-term safety data. Additionally, compensatory pathways may render them ineffective, thus necessitating the development of therapies that block several immune responsiveness pathways.The interaction of IL-17A with IL-17RA is approximately 100-fold stronger than that of IL-17F with IL-17RA, as shown by key residues at the interface. Rational mutagenesis of selected residues (R55V, W67V, Y85I, H86S), which are critical for the maintenance of the stability of the complexes. Liu et al pointed out that site-specific mutations of the above-listed residues that correspond to those found in IL-17A mimic the interaction strengths in IL-17A [34].

An atomistic understanding of protein–protein interactions remains underexplored but is requiredfor tuning binding energetics and target specificity to perturb macromolecular interactions. The newly developed methods are crucial to be able to reconfigure the network of interactions between molecules. In silico strategies are key for the identification of critical hotspot residues at the interface, as illustrated in previous studies by Wells et al., who showed that certain key residues can have a significant effect on the thermodynamic stability of the binding. Single-point mutations are also shown to affect the structure by alanine mutagenesis to identify hotspots [35,36]. Such types of computational tools can offer markers of the site-specific inhibitors to regulate the interactions underlying therapeutic potential.

To investigate the energetic features and structural variability of IL-17A/IL-17RA interactions, we use advanced computational approaches such as site-specific mutagenesis and simulation-based analyses. In silico mutational analysis offers a quick, cheap, and powerful method to explore the consequences of amino acid changes on protein and biological activity when compared to traditional in vitro approaches. To overcome kinetic trapping and ensure adequate sampling of conformational space, multiple replica molecular dynamics (MD) simulations have emerged as a robust strategy to validate thermodynamic stability and structural transitions. Recent studies have further highlighted the value of multiple-replica simulations in biomolecular systems [37,38]. Consequently, conducting replica simulations provides enhanced statistical convergence when evaluating mutational impacts on biomolecular complexes. By focused amino acid substitutions, the IL-17A residues were swapped with those of IL-17F, revealing the structural mechanisms that underlie the recognition and selectivity of the cytokine–receptor interaction. Such a computational paradigm is not only useful for traditional drug development pipelines but also expands the underlying scientific mechanisms and elucidation of protein–protein interface perturbation by mutations. Altogether, our study provides the basis for rational design of therapeutic inhibitors and new opportunities for therapeutic intervention in autoimmune diseases and IL-17A associated cancers.

## 2. Methodology

### 2.1. Structure retrieval preparation; IL-17A/IL-17RA complex

The co-crystal structure of the IL-17A homodimer bound to the extracellular D1:D2 domains of the IL-17RA receptor (PDB ID: 4HSA), determined by X-ray crystallography at 3.15 Å resolution, was obtained from the Protein Data Bank [34]. This structure includes the IL-17A homodimer and the IL-17RA receptor chain, with a total of 527 amino acids. The D1 and D2 domains of IL-17RA are members of the type-III fibronectin family [24], with D1 binding both monomers of IL-17A, while D2 only binds one monomer [39]. Gaps in the IL-17A structure, comprising residues Pro11-Lys16 at the N-terminus and Asn30-Ser40 in the internal loop, were modelled using Modeller (v10.0) [40]. The preprocessing steps were carried out in the Molecular Operating Environment (MOE) v2019.01 [41] and included the following modifications: removal of unwanted groups from the molecule; setting the bond orders dependent on chemical environment; and making formal charges on ionizable residues correct. Hydrogen atoms were added using MOE’s Protonate 3D algorithm, which ensured hydrogen bonds were optimized. Partial charges of atoms were obtained by using the AMBER99 force field. To model a physiologically relevant aqueous environment, protonation states of titratable residues were determined using MOE’s electrostatic function, which uses the generalized Born/volume integral (GB/VI) implicit solvent model to calculate the pKa values at physiologically relevant pH (pH 7.0). The contribution of interaction forces between the molecules was evaluated as intermolecular non-bonded forces using a 15 Å interaction cutoff for inclusion of long-range electrostatic forces and a 10 Å cutoff radius for van der Waals short-range interactions. The minimization of the system was performed at a default RMS gradient of 0.1 kcal·mol-1·Å-2 with harmonic restraints (1000 kJ·mol-1·nm-2) on backbone atoms to ensure the integrity of the system.

### 2.2. Prediction tools for analyzing mutation impacts

A wide computational framework was used to evaluate the effect of amino acid changes on protein function based on intermolecular binding affinity and protein structural stability. Alterations in binding affinity were calculated using DDMut-PPI [42], MCSM-PPI2 [43], and BeAtMuSiC [44], which estimate changes in protein binding free energy (ΔΔG) to characterize the effects of mutations on binding. To assess the possible stabilizing or destabilizing effects of these mutations on protein conformation, CUPSAT was used to determine the changes in protein stability (ΔΔG) in the mutant structures [45]. Also, the DUET method was used, which is based on averaging several algorithms to achieve a better accuracy [46]. The MOE program suite allowed for advanced modeling and simulation for further detailed structural and stability analysis [41]. The effect of mutations on protein dynamics was also explored with DynaMut [47], and further insight into differences in binding energies was obtained with SAAMBE [48]. These tools provided a comparative analysis of the impact of mutations in Chains A and B, particularly the substitutions of corresponding residues from IL-17F for R55V, W67V, Y85I, and H86S in IL-17A (Table 1 and S1 Fig in S1 File). The inferred change in binding and stability of the mutant (MT) and wild-type (WT) protein complexes was used to evaluate the effect of these mutations. The ΔΔG values are the difference in binding energy of the MT and WT complexes and indicate the effect of the mutation:

**Table 1:** Key interfacial hotspot residues selected for in silico mutagenesis. IL-17A Pocket Mutant Residues, Types, Positions, Nature of Substitutions and Mutation Codes.

Wild Type	Code	Nature	Position	Mutant Type	Code	Nature
Arginine (Arg)	R	Basic	**55**	Valine (Val)	V	Non-Polar
Tryptophan (Trp)	W	Non-Polar	**67**	Valine (Val)	V	Non-Polar
Tyrosine (Tyr)	Y	Non-Polar	**85**	Isoleucine (Ile)	I	Non-Polar
Histidine (His)	H	Basic	**86**	Serine (Ser)	S	Polar


$$\begin{array}{c} \Delta \Delta G_{WT-MT}=\Delta G_{wild-type}-\Delta G_{Mutant-type} \end{array}$$ (1)

### 2.3. Computational mutagenesis

The effects of amino acid substitutions on the structure and binding affinity of IL-17A with its receptor IL-17RA were evaluated with computational mutagenesis in UCSF Chimera 1.14 [49]. The preprocessed energy-minimized structure of IL-17A was taken as a reference. Using the results from prediction tools that evaluate the effect of amino acid mutations, residues with the strongest effect on protein-protein interactions were chosen. The mutations were selected to reverse the destabilizing influence seen in the complex. In particular, residues Y85I and H86S in Chain A, and R55V and W67V in Chain B, were substituted with their counterparts from IL-17F (Fig 1). The “Rotamer” feature in Chimera was used to adjust side chains to avoid steric clashes while preserving reasonable conformations. Energy minimization of the mutants was performed using the AMBER99 force field to remove steric clashes and produce stable models.

**Fig 1:**
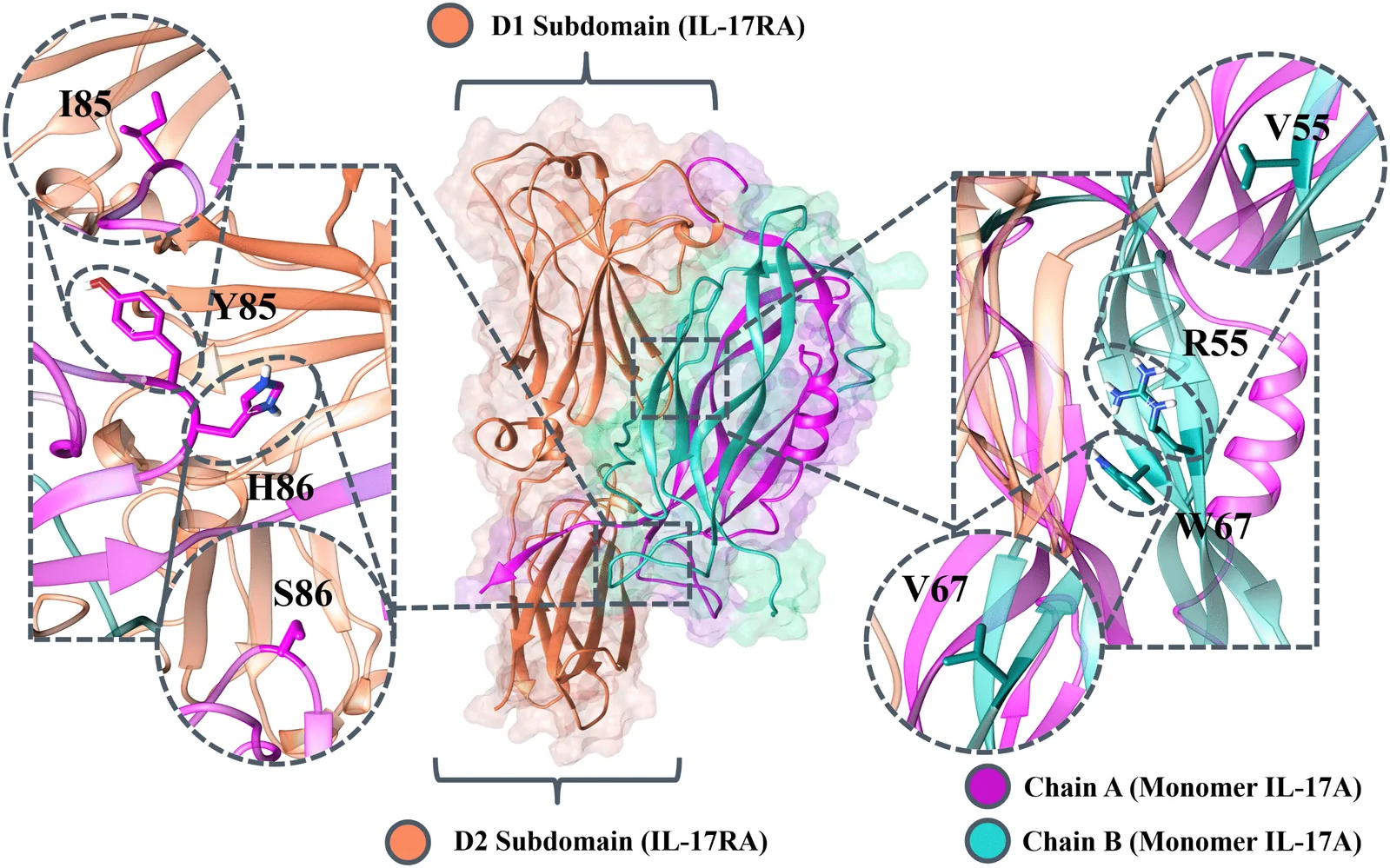
Structural architecture of the IL-17A/IL-17RA complex. Ribbon representation of the IL-17A/IL-17RA complex with the different domains: subdomain 1 and subdomain 2 (salmon), IL-17A Chain A (purple) and IL-17A Chain B (cyan). Residues at mutational sites are marked and depicted in grey sticks.

### 2.4. Molecular dynamics simulation

To assess the structural and functional implications of the IL-17A/IL-17RA wild-type (WT) protein and its four mutants (R55V, W67V, Y85I, and H86S), extensive molecular dynamics (MD) simulations were performed. The simulations were run with GROMACS 2022.3 using CUDA acceleration on an NVIDIA GTX 1070 graphics processing unit (GPU) [50]. The pdb2gmx program was used to assign the protonation states of the titratable residues at pH 7.0 upon the addition of all missing hydrogens. Histidine residues were automatically assigned to the HID/HIE tautomer according to the force-field specifications, while Asp, Glu, Lys and Arg were protonated according to their ionization state at a neutral pH. This ensured chemically correct protonation prior to solvation and MD simulations. Topology files were prepared for all systems using the Amber ff99SB‐ILDN force field [51]. The protein configurations were centered in a 10 × 10 × 10 nm cubic box with a 1.0 nm buffer between the protein and the box walls. TIP3P water molecules were added to solvate the system, ensuring a density below 1000 g/cm^3^ [52]. Counter-ions (Na+) were introduced using the GROMACS genion tool, substituting water molecules to neutralize the system’s net charge. Periodic boundary conditions were employed to avoid boundary artefacts. A steepest descent minimization algorithm optimized the system for 50,000 steps, followed by a two-phase equilibration with heavy atom constraints. The first phase involved 100 ps of heating in the NVT ensemble, gradually increasing the temperature from 0 K to 300 K using a velocity-rescale thermostat. The second phase consisted of equilibration within the NPT ensemble at 1 bar for 500 ps to 2 ns, allowing thermal and pressure stabilization. Temperature and pressure were regulated using the velocity-rescale thermostat and the Parrinello-Rahman barostat [53,54]. The production MD simulations lasting 200 ns were conducted on each of the five systems, which include wild-type and four mutants, along with their corresponding replicates. Each system was simulated three times using independent initial velocity assignments to ensure reproducibility of dynamic behavior. Significant insights into mutation-induced structural dynamics and stability have been obtained from a total simulation period of 3 µs at constant temperature and pressure, removing atomic restraints and allowing free atom movement in a solvated system. This setup allowed sufficient conditions to simulate structural and dynamic properties. Global structural descriptors, including the radius of gyration (Rg), root mean square deviation (RMSD), and root mean square fluctuation (RMSF), which were calculated separately for each replicate. The mean value obtained from each independent trajectory was treated as a single observation, and the results are reported as mean ± standard deviation (SD) across the three independent simulations. Meanwhile, the hydrogen bonding network has been systematically observed during the molecular dynamics simulations. To capture the temporal stability and structural shifts of the system, key MD trajectory parameters, including root-mean-square deviation (RMSD), root-mean-square fluctuation (RMSF), and hydrogen-bond counts over time were extracted and plotted as a function of simulation time (ns) using Xmgrace [55]. Detailed structure analysis and visualization were performed with Visual Molecular Dynamics (VMD) [56]. Hydrogen-bond interactions were analyzed using the built-in HBonds plugin in VMD with a donor–acceptor distance cutoff of 3.5 Å. The VMD HBonds plugin calculates the occupancy of each donor–acceptor atom pair over the trajectory. For residue-level analysis, the occupancies of all distinct donor-acceptor pairs involving the residue were summed to obtain the cumulative residue-level hydrogen-bond occupancy. Principal Component Analysis (PCA) was performed to characterize the conformational space, and Free Energy Landscape (FEL) plots were employed to analyze the energy landscape of the system.

### 2.5. Protein structure network dynamics

Protein Structure Network (PSN): A computational analysis tool for the structural topology and functional relationships within proteins. Produces network representations with residues as nodes and non-bonded interactions as links in the network. The analysis provides valuable information on macromolecular stability, folding pathways, molecular recognition and allosteric modulation [57,58]. To explore the network of interactions among the WT and MT protein systems, the PSN analysis was performed using the WebPSN 2.0 server [59], which is an advanced tool to process the molecular dynamics trajectories and structural information in 3D. The structural properties of the systems were analyzed using key parameters of the network such as the minimum interaction strength (Imin) that determines the node connectivity, the hubs (residues with ≥4 connections), the communities (clusters of highly connected residues), and the shortest path (communication efficiency) [60]. Networks of structures were constructed from simulation data at 100 ns intervals, with representative conformations being extracted. With the assembly of data from the conformational ensemble, the “Consensus PSN” tool was used to detect stable structural communities. The “PSN Difference” module was also used to detect changes in network topologies between targeted systems by detecting changes in critical residues and their patterns of connectivity. By applying PSN analysis to MD simulation trajectories, we captured the impact of point mutations on structural communication and stability within the protein.

### 2.6. Principal Component Analysis (PCA) and Free Energy Landscape (FEL)

Principal Component Analysis was used to investigate the dynamic and structural properties of the IL-17A/IL-17RA protein and assess the effect of mutations. PCA is a widely used statistical technique for dimensionality reduction that preserves the most important features of high-dimensional data [61,62]. The technique enhances the interpretability of molecular simulations by extracting the underlying collective dynamics. Empirical studies use PCA in conjunction with FEL analysis to enhance understanding of conformational dynamics and thermodynamic properties. In the present study, PCA on the backbone atoms for both WT and MT systems has been performed using the MD analysis tools [63,64]. FEL analysis was done using the GROMACS program gmx sham, which computes ΔG as a function of the first two principal components (PC1 and PC2) [65]. Gibbs free energy was computed using the following formula:


$$\begin{array}{c} \Delta G=-k_{B}TlnP\;\left(PC1,PC2\right) \end{array}$$ (2)

Here, T is the absolute temperature, kB is the Boltzmann constant, and P(PC1, PC2) is the probability distribution of the system along principal components PC1 and PC2. The results of this study shed light on the impact of mutations on the thermodynamics and conformational dynamics of the protein by delineating distinct energy landscapes for the studied systems.

### 2.7. Comprehensive analysis of the Dynamic Cross-Correlation Matrix (DCCM)

To reveal the correlated motions and system dynamics, the Dynamic Cross-Correlation Matrix (DCCM) analysis was performed in Bio3D running in RStudio [66,67]. Trajectory frames from the 50–100 ns time window were used for the analysis of the atomic displacement correlations. For enhanced accuracy of results and damping statistical noise, Cα atomic coordinates alone were employed in DCCM analysis to achieve a better estimate of inter-residue correlations and dynamic contacts.

### 2.8. Molecular Mechanics/Generalized Born Surface Area (MM/GBSA) energy calculation

For the determination of binding free energies for MT and WT complexes, the Molecular Mechanics/Generalized Born Surface Area (MM/GBSA) protocol was applied through the HawkDock web server. HawkDock is an easy and robust computational platform for predicting and analyzing protein-protein complexes due to its optimized support for per-residue decomposition; however, it does not provide error estimations for the calculated binding energies [68]. The approach combines molecular docking with MM/GBSA-based binding free energy calculation. To account for conformational fluctuations during the MD simulations, MMGBSA binding free energies were calculated using five representative structures extracted from MD trajectories at 0, 50, 100, 150, and 200 ns. For each representative structure, the van der Waals energy (ΔE_VDW_), electrostatic energy (ΔE_ELE_), polar solvation energy (Δ_GGB_), non-polar solvation energy by solvent-accessible surface area (∆G_SA_), and total binding free energy (∆G_MMGBSA_) were computed. The binding free-energy components obtained from the five representative structures were averaged and are reported as the mean ± standard deviation (SD). This ensemble-based approach provides a more reliable estimate of the relative binding affinities than a single-frame analysis. To evaluate the asymmetric dimeric interfaces independently, MM/GBSA calculations were performed separately for Chain B with Chain C (WT, R55V, and W67V) and Chain A with Chain C (WT, Y85I, and H86S). The MMGBSA calculations were performed by ff02 force field, which is the default force field implemented in the HawkDock server, and omits conformational entropy contributions. Energy minimization was performed to optimize the structures using 2000 cycles of steepest descent and 3000 cycles of conjugate gradient. This is an automated procedure that guarantees that the reduced complexes are structurally stable and correct for further free energy calculations [68]. Essential residues that play a crucial role in protein–protein interaction were identified through residue-wise decomposition analysis.

## 3. Results and discussion

Coordinated protein–protein interactions regulate biological mechanisms such as metabolism, intracellular signaling and molecular transport. Interactions are mediated by critical hotspot residues at protein–protein interfaces, and mutations in these areas tend to compromise the function of proteins. Liu et al. showed that making specific mutations to the IL-17A sequence, such as replacing amino acids with their counterparts in IL-17F, affects the ability of the receptor to bind to the cytokine and how it interacts with downstream functional partners. They performed site-directed mutations to determine that residues R55, W67, Y85 and H86 are essential for the function of IL-17A and thus essential for further mechanistic studies. Considering the time-consuming nature of in vitro characterization experiments, here, we employed computational mutagenesis to investigate the effects of mutating these residues.

### 3.1. Computational mutagenesis

Mutagenesis studies play a crucial role in investigating structural and functional changes in PPIs. We demonstrate the critical contribution of hotspots (R55, W67, Y85, and H86) in the stability and function of the IL-17A/IL-17RA interaction. Analysing the variable effects of mutations in Chain A and Chain B provided insight into the contribution of particular amino acids to these interactions. ΔΔG values, calculated from physicochemical properties, were employed to determine the destabilizing or stabilising effects of mutations. Online tools allowed the real-time assessment of PDB structures and mutations, serving as the foundation of our mutational analysis. These web-based prediction tools provide deterministic estimates from static PDB structures used as an initial consensus screen to identify key interface mutations and establish a structural baseline before long-timescale molecular dynamics (MD) simulations. Chain-specific mutations of IL-17A had varying impact on binding affinity, hinting at possible therapeutic approaches to modulate IL-17A receptor assembly and inflammation.

#### 3.1.1. Comprehensive mutational profiling and impact on binding affinity.

The effects of mutations on binding affinity were calculated using MOE and machine learning tools such as DDMut-PPI, mCSM-PPI2, and BeAtMuSiC (Fig 2 and S1 Table in S1 File). The variations in the calculated ΔΔG values might be due to the different algorithms and scoring methods used, which represent single-structure deterministic predictions without statistical sample variance. DDMut-PPI and MCSM-PPI2 define negative ΔΔG values as decreases in binding affinity, and BeAtMuSiC and MOE consider negative and positive values as stabilizing and destabilizing effects, respectively. Mutations in Chain B, such as R55V and W67V, are important for determining binding affinity and macromolecular association. Calculations using DDMut-PPI and BeAtMuSiC revealed that the R55V mutation in Chain A had a slight effect on binding affinity, −0.097 kcal/mol and 0.01 kcal/mol, respectively. On the other hand, MCSM-PPI2 and MOE reported a slight increase in binding affinity. In contrast, the same R55V mutation in Chain B exhibited a significant destabilizing effect by the BeAtMuSiC (−0.10 kcal/mol), MOE (0.418 kcal/mol), DDMut-PPI (−0.527 kcal/mol) and MCSM-PPI2 (0.233 kcal/mol), highlighting the role of this mutation in protein stability. W67V was the most destabilizing especially in Chain B. Although in Chain A some decrease in binding affinity was observed (DDMut-PPI: −0.028 kcal/mol, MCSM-PPI2: −0.109 kcal/mol, BeAtMuSiC: 0.23 kcal/mol), the effect was more pronounced in Chain B. W67V in Chain B had a dramatic effect, with DDMut-PPI (−1.146 kcal/mol), MCSM-PPI2 (−2.044 kcal/mol), MOE (1.366 kcal/mol) and BeAtMuSiC (2.68 kcal/mol), implying a major destabilization effect in protein-protein interactions. Moreover, Y85I and H86S mutations in Chain A had profound effects on binding affinity. Y85I and H86S in Chain A showed destabilizing effects (BeAtMuSiC: ΔΔG values of 1.06 kcal/mol for Y85I and 1.11 kcal/mol for H86S), which were confirmed by MOE (ΔΔG: 0.478 kcal/mol for H86S and 0.268 kcal/mol for Y85I). Their impact on Chain B was limited, with DDMut-PPI and BeAtMuSiC reporting either slight decreases or increases in binding affinity. These results reveal the importance of residues Y85 and H86 in Chain A, whose mutations strongly hinder receptor binding. The overall destabilizing effect of W67V in Chain B also underlines its role in the stability of the IL-17A/IL-17RA interaction. The values reflect deterministic algorithm outputs derived from static complex coordinates. This computational mutagenesis analysis served as an initial consensus-based screen to identify key interface residues, whereas the subsequent multi-replicate molecular dynamics simulations provide a full, ensemble-based assessment of the structural and dynamic consequences of the selected mutations. Overall, the knowledge of residue-specific interactions will guide the design of drugs that bind to the receptor complex.

**Fig 2:**
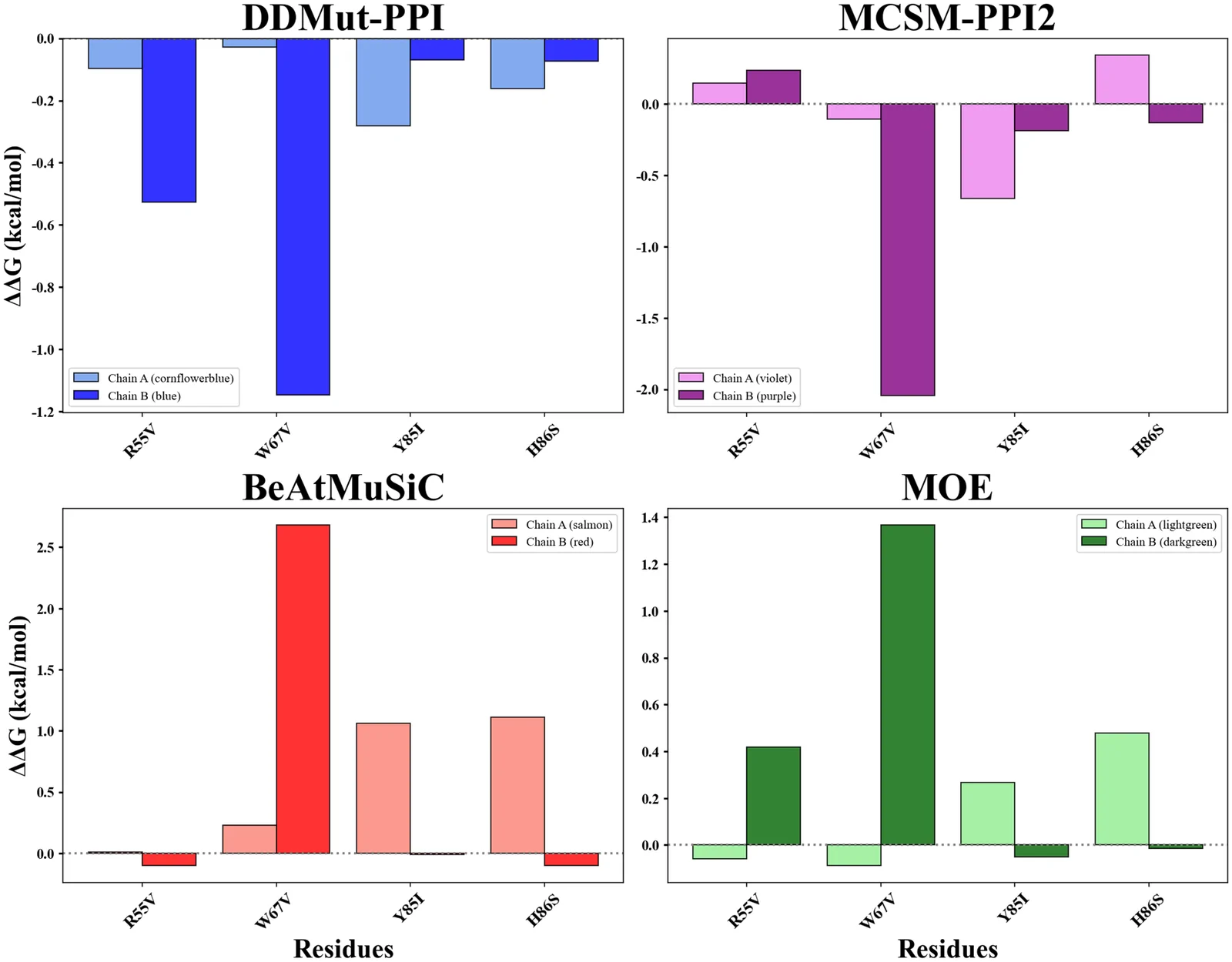
In silico mutational impact on IL-17A/IL-17RA intermolecular binding affinity. Binding energies of IL-17A/IL-17RA pocket residues from static frame deterministic scoring algorithms, predicted by different computational programs. The x-axis labels mutated residues, with the y-axis displaying binding energies (kcal/mol). Chain B residues are shown in dark colors, while Chain A residues are in a lighter shade.

#### 3.1.2. Predictive modeling of mutational impacts on IL-17A dimer stability.

The effects of mutations (R55V, W67V, Y85I, and H86S) on the stability of the IL-17A homodimer were predicted by CUPSAT, DUET, DynaMut, and SAAMBE, and the results are shown in Fig 3 and S2 Table in S1 File. Importantly, SAAMBE considers negative ΔΔG values as a predictor of stabilization, while CUPSAT, DUET, and DynaMut consider negative ΔΔG as destabilizing. For the R55V mutation, Chain A showed conflicting predictions: CUPSAT (−1.68 kcal/mol) and SAAMBE (0.57 kcal/mol) showed destabilization, while DUET (0.237 kcal/mol) and DynaMut (0.13 kcal/mol) showed slight stabilization. In contrast, Chain B consistently showed destabilization, with ΔΔG values of −0.67 kcal/mol (CUPSAT), −0.019 kcal/mol (DUET), −0.36 kcal/mol (DynaMut), and 0.85 kcal/mol (SAAMBE). The W67V mutation demonstrated conflicting stability predictions, with CUPSAT identifying stabilization (1.46 kcal/mol) in Chain A, while DUET (−1.214 kcal/mol), DynaMut (−1.46 kcal/mol), and SAAMBE (0.23 kcal/mol) reported destabilization. Unlike Chain A, Chain B was consistently predicted to be destabilizing, to varying degrees, including DUET (−2.491 kcal/mol), DynaMut (−2.78 kcal/mol), SAAMBE (1.34 kcal/mol), and CUPSAT (0.63 kcal/mol). The Y85I mutation in Chain A showed a significant destabilising effect ranging from ΔΔG = −1.26 (CUPSAT) to −1.09 (DynaMut) with SAAMBE reporting 1.51. Chain B showed a low level of destabilization based on the DUET (−0.351 kcal/mol), DynaMut (0.06 kcal/mol), and SAAMBE (1.09 kcal/mol) methods, while CUPSAT predicted a much larger destabilizing effect (6.48 kcal/mol). The H86S mutation also showed chain-specific differences. In Chain A, DUET (0.525 kcal/mol) and DynaMut (1.75 kcal/mol) predicted stabilization, while CUPSAT (−2.58 kcal/mol) and SAAMBE (0.01 kcal/mol) predicted destabilization. However, in Chain B, all tools predicted stabilization, with DUET (0.384 kcal/mol) and DynaMut (1.79 kcal/mol) showing similar results, while CUPSAT (6.02 kcal/mol) and SAAMBE (−0.14 kcal/mol) also indicated stabilization. A combination of mutational prediction results and PPI analysis from PDBsum (S2 Fig in S1 File) [69], identified the residues R55 and W67 (chain B, D1 subdomain) and Y85 and H86 (chain A, D2 subdomain) as hotspots at the interface whose mutations destabilize the IL-17A homodimer [70].

**Fig 3:**
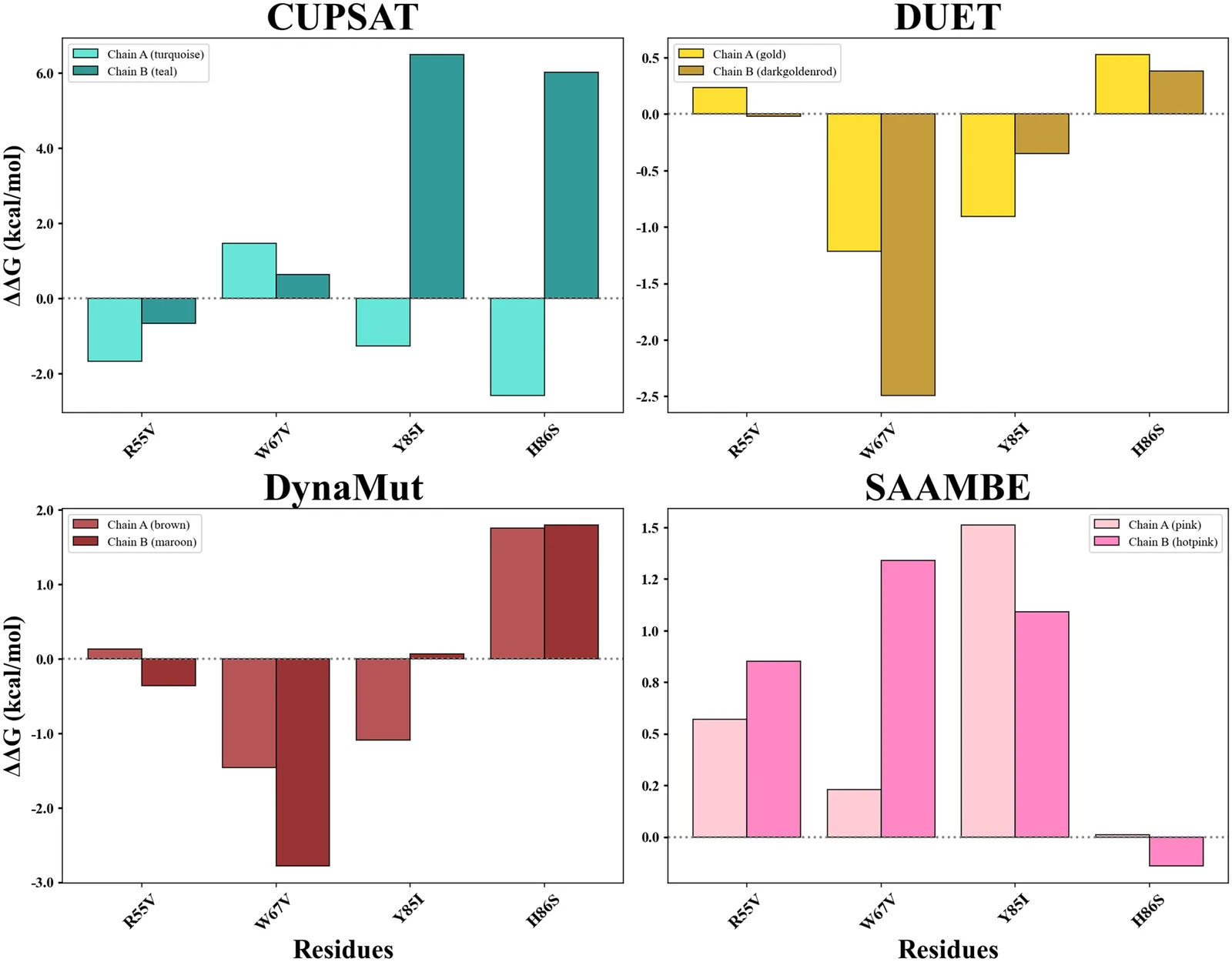
Predicted impact of hotspot point mutations on structural stability. Deformation energy (kcal/mol) of the WT and four single-point mutations at the interface of IL-17A/IL-17RA, computed by various servers. The x-axis represents the mutated residues, and the y-axis represents the energy. The darker bars represent Chain B residues and the lighter bars represent Chain A residues.

### 3.2. Molecular dynamics simulations reveal mutation-induced alterations in IL-17A dynamics

Molecular dynamics simulations were conducted to explore the effect of R55V, W67V, Y85I, and H86S mutations on the structural and dynamic properties of the IL-17A protein to understand the mutation-induced changes in its stability and flexibility that may affect its binding to the IL-17RA receptor. MD simulations of the WT and MT systems were carried out using all-atom models, and trajectory analyses using RMSD, RMSF, and Rg were used to investigate the stability and flexibility of the systems. Dynamic stability was assessed by the root mean square deviation (RMSD) of backbone atoms (N, Cα, C, and O) from their initial structure for 200 ns (Fig 4). The RMSD curve of the WT complex remained stable after ~130 ns and the mean RMSD was 0.264 ± 0.028 nm, indicating stabilization of the structure during the simulation. The mutant complexes generally exhibited slightly higher RMSD values than the WT; however, the differences remained modest, and all systems reached stable trajectories during the simulations. Most noticeably, MT R55V exhibited a relatively high RMSD value of 0.299 ± 0.031 nm, meaning that it was more flexible and had structural perturbations relative to the WT. The type of mutation W67V is showing RMSD value very close to WT, indicating this mutation has little effect on backbone atoms deviation. Y85I exhibited a modest increase in RMSD (0.306 ± 0.051 nm) compared with the WT, indicating slightly greater conformational fluctuations during the simulation. H86S mutation had RMSD value of 0.278 ± 0.031 nm, which is intermediate between MT R55V/Y85I and WT and which resulted in minute structural changes. These RMSD analysis results show that the mutations R55V and Y85I display modestly higher backbone fluctuations in the structure of IL-17A, which could influence its ability to bind to the receptor IL-17RA.

**Fig 4:**
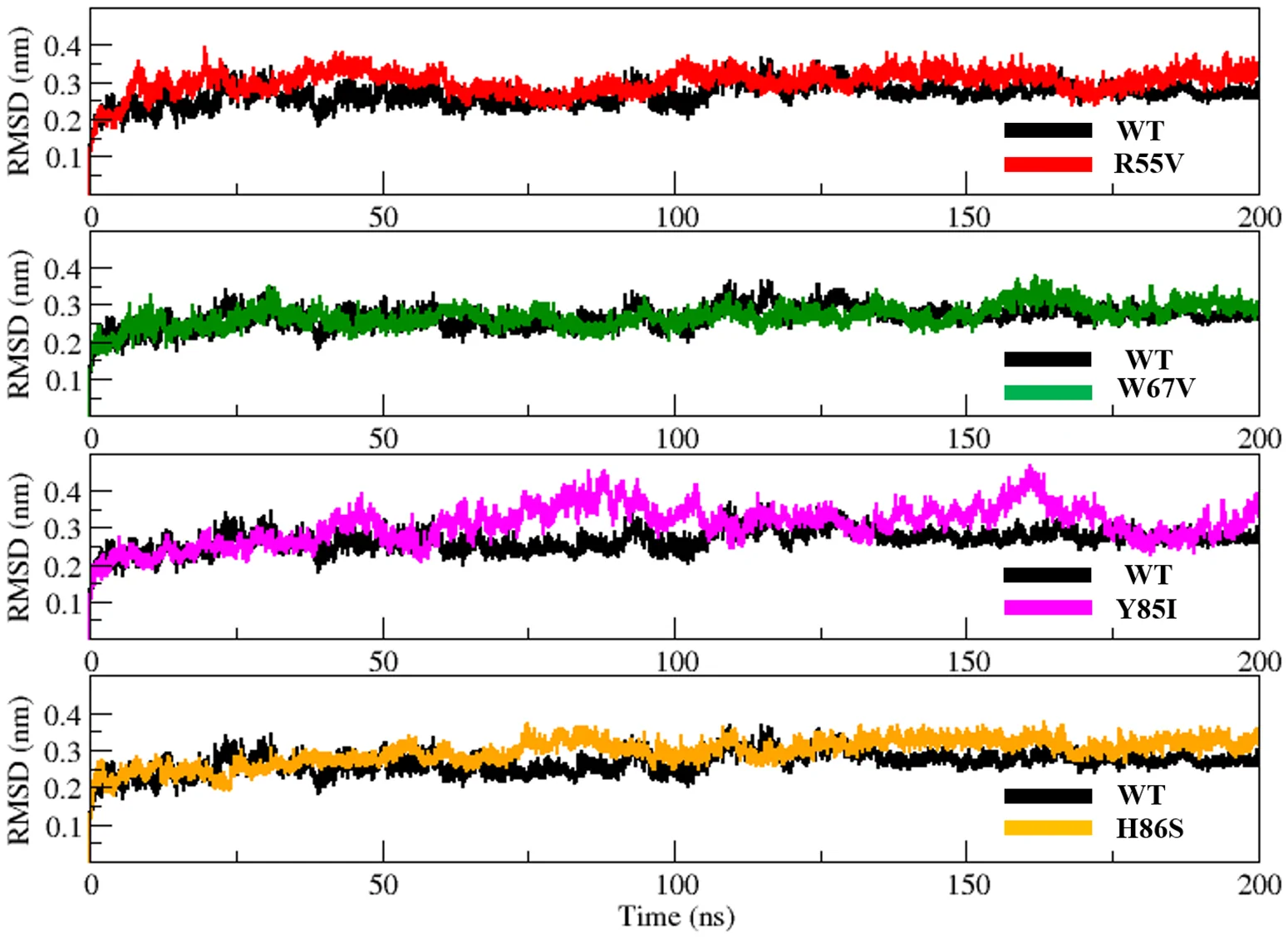
Temporal backbone RMSD trajectories across WT and variant complexes. Dynamic behavior of WT (black) and MT (R55V, red; W67V, green; Y85I, magenta; H86S, orange) systems over 200 ns, indicating conformational flexibility of the protein.

To further assess the reproducibility of the RMSD behavior, two additional independent 200 ns MD replicas (R2 and R3) were performed for the WT and mutant IL-17A systems. Comparison across all three independent replicas showed consistent overall RMSD behavior with relatively low inter-replica variability. As shown in Fig 5, the mean RMSD values obtained from replicates 1–3 remained comparable across the independent simulations, indicating reproducible structural behavior. Specifically, the WT system exhibits stable mean RMSD values of 0.26 ± 0.031 nm, 0.25 ± 0.031 nm, and 0.26 ± 0.031 nm across replicates 1, 2, and 3, respectively. Similarly, the variant systems display close structural agreement among replicate runs, confirming that the observed conformational behaviors reflect true system equilibrium rather than stochastic sampling artifacts. The RMSD profiles of the independent replicate simulations (Replicates 2 and 3), are presented in S3 and S4 Figs in S1 File, respectively. A compiled comparison of the RMSD profiles from all three replicas is shown in S5 Fig in S1 File. The corresponding time-averaged RMSD values, reported as mean ± SD for each replica, are summarized in S3 Table in S1 File.

**Fig 5:**
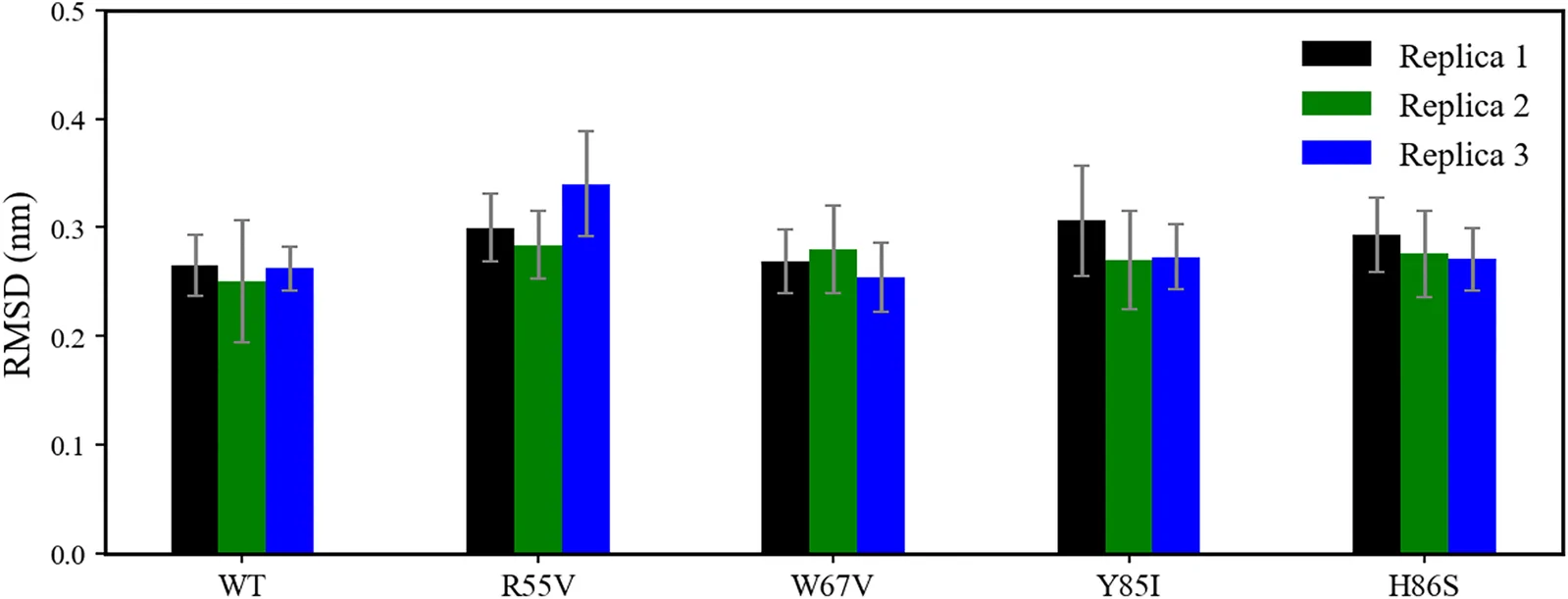
Replicate-averaged backbone RMSD comparisons. RMSD values (Mean±SD) across three independent simulation replicas (Replica 1, Replica 2, and Replica 3) for WT and mutant complexes (R55V, W67V, Y85I, and H86S).

In order to elucidate the flexibility at residue level, the fluctuation of the backbone atoms was performed by RMSF analysis. The higher the RMSF values, the greater the conformational flexibility and the greater the inferred instability [71]. Variability patterns among variants (WT and MT) are given in Fig 6. The average RMSF values were calculated as follows: WT (0.138 ± 0.104 nm), R55V (0.123 ± 0.083 nm), W67V (0.140 ± 0.116 nm), Y85I (0.148 ± 0.116 nm), and H86S (0.146 ± 0.111 nm). Of particular interest is that the average RMSF for R55V was slightly lower than that for the WT, suggesting lower atomic mobility. In contrast, the other MT (W67V, Y85I and H86S) had higher RMSF value, which represents higher residue flexibility. Relatively larger fluctuations were observed in residues 430−500 of Chain C, particularly in W67V and Y85I mutant complexes, relative to WT, representing allosterically induced flexibility within the receptor loops. These differences suggest localized changes in residue flexibility that may influence the interaction with IL-17RA.

**Fig 6:**
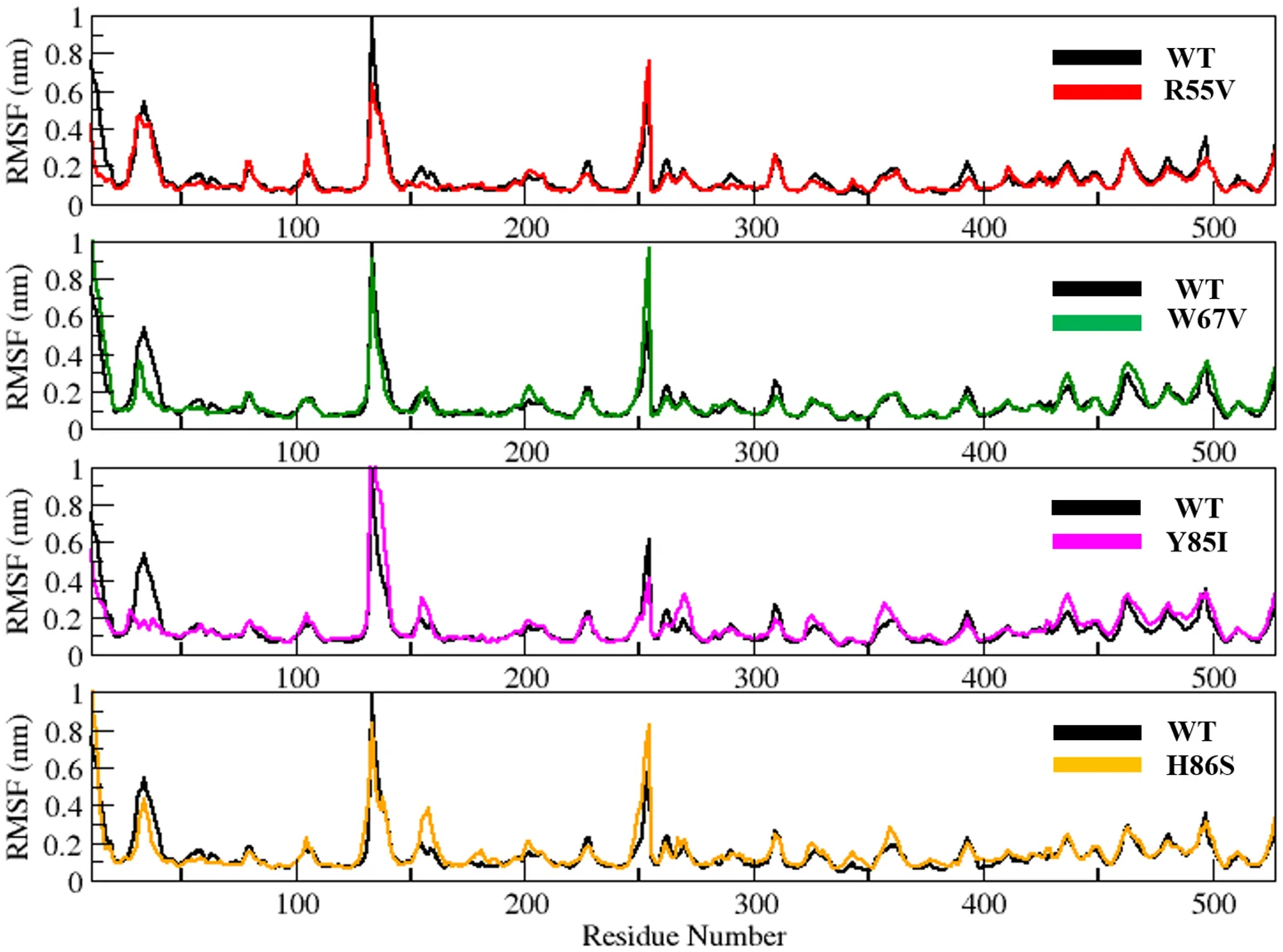
Dynamic variations in residues of IL-17A. The graph shows the Per-residue backbone RMSF profiles along the wild-type (black) and mutant (R55V, red; W67V, green; Y85I, magenta; H86S, orange) IL-17A structures.

To evaluate local backbone mobility across independent simulation trajectories, RMSF values were compared across all three MD replicates for WT and variant complexes. As shown in Fig 7, the average residue fluctuations remain highly consistent across Replicates 1, 2, and 3, demonstrating excellent reproducibility. For the WT system, mean RMSF values across all residues were 0.138 ± 0.105 nm (Replicate 1), 0.142 ± 0.106 nm (Replicate 2), and 0.160 ± 0.088 nm (Replicate 3). The mutated complexes displayed similarly consistent mobility patterns, with standard deviations reflecting intrinsic differences between rigid secondary structures and flexible loops rather than run-to-run variation. Overall, these results confirm that local dynamic behavior is preserved across independent replicates. The RMSF profiles of the independent replicate simulations (Replicates 2 and 3), are presented in S6 and S7 Figs in S1 File, respectively. A compiled comparison of the RMSF profiles from all three replicas is shown in S8 Fig in S1 File. The corresponding residue-wise RMSF values, reported as mean ± SD for each replica, are summarized in S4 Table in S1 File.

**Fig 7:**
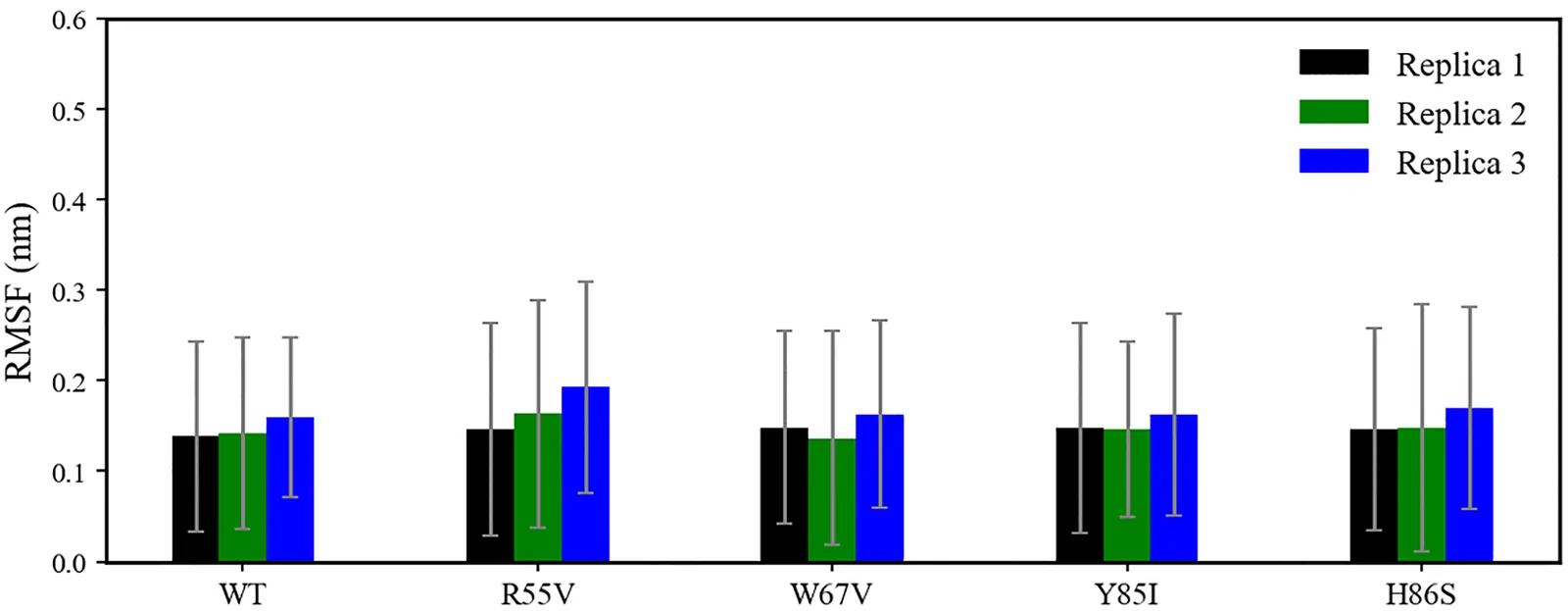
Residue flexibility across multi-replicate MD runs. Comparison of mean per-residue RMSF values (Mean±SD) across three independent simulation replicas (Replica 1, Replica 2, and Replica 3) for WT and mutant complexes.

The dynamic changes and variations in the structures observed in the simulations were also investigated through the gyration pattern. The Rg is a measure of the compactness and stability of a protein in solution and is the mass-weighted root-mean-square distance of atoms from the protein’s centre of mass. Constant Rg values typically indicate a well-folded and stable protein [72]. The Rg of the WT and MT systems was calculated during the simulation to observe the effects of the mutations on protein stability and compactness (Fig 8). The Rg values for the W67V (2.721 ± 0.017 nm) and H86S (2.725 ± 0.013 nm) mutants showed marginal increases compared to the WT (2.712 ± 0.014 nm). By contrast, the Rg of the R55V (2.748 ± 0.016 nm) and Y85I (2.747 ± 0.019 nm) mutants exhibited higher Rg values than WT, indicating a slight reduction in protein compactness. The observed differences were relatively small and are more consistent with modest changes in conformational flexibility than with global protein unfolding. Overall, the Rg analysis suggests that R55V and Y85I exhibit a tendency toward reduced compactness relative to the WT, whereas W67V and H86S remain largely comparable to the WT.

**Fig 8:**
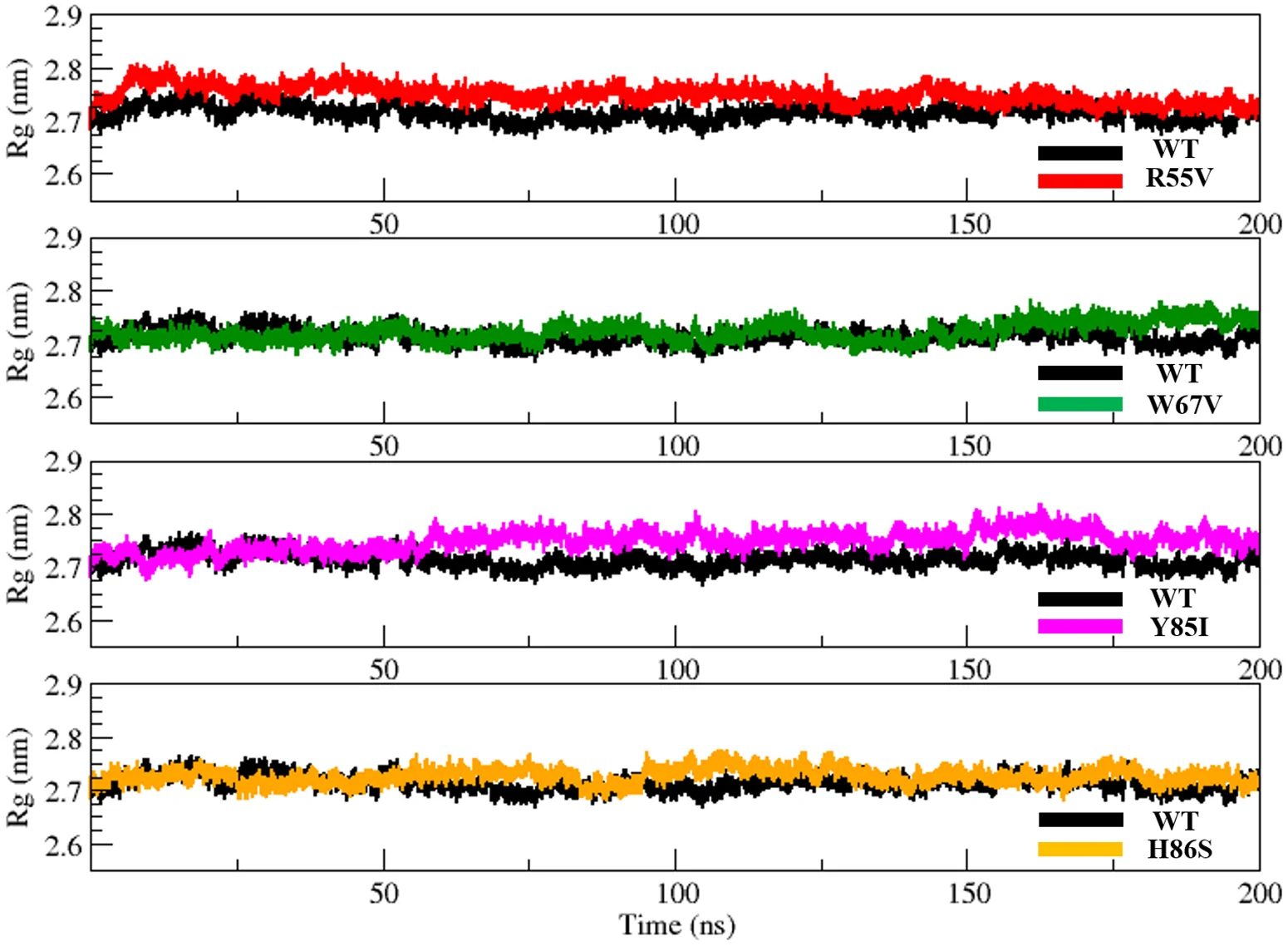
Compactness of IL-17A measured by Radius of gyration (time-dependent). The Fig displays an Rg plot of the wild-type and mutant structures of IL-17A, which shows the changes in dimension that occurred during the simulation time.

Rg values were evaluated across all three MD replicates for WT and variant complexes. As shown in Fig 9, the average Rg values exhibit exceptional consistency across Replicates 1, 2, and 3, confirming that overall tertiary structural compactness is stably maintained across independent runs. For the WT system, mean Rg values were 2.713 ± 0.014 nm (Replicate 1), 2.697 ± 0.023 nm (Replicate 2), and 2.707 ± 0.015 nm (Replicate 3). The mutated complexes displayed similarly uniform values, with minor fluctuations falling well within standard thermal variation, confirming the absence of major structural expansion or unfolding events across replicate runs. The Rg profile of replicates 2 and 3 is presented in S9 and S10 Figs in S1 File, respectively. A compiled comparison of the Rg profiles from all three replicas is shown in S11 Fig in S1 File. The corresponding time-averaged Rg values, reported as mean ± SD for each replica, are summarized in S5 Table in S1 File.

**Fig 9:**
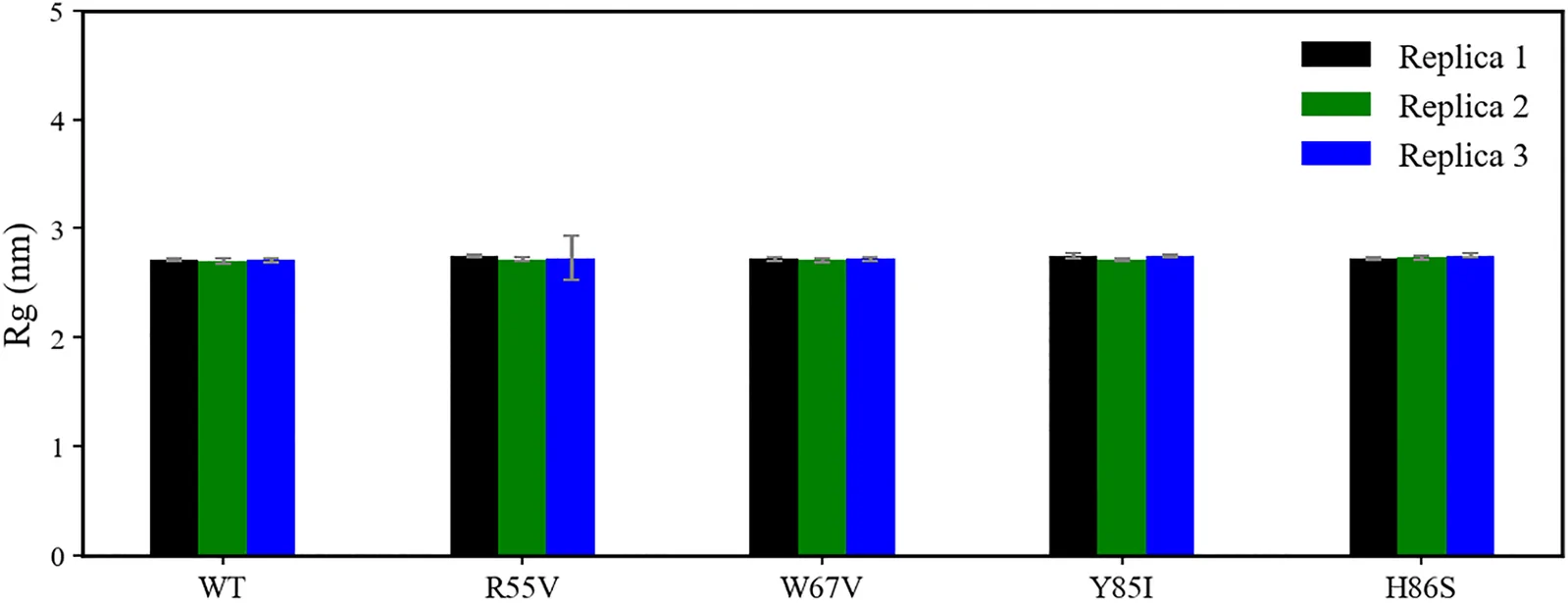
Mean radius of gyration (Rg) values across three independent MD simulation replicas for Wild-Type and variant complexes.

Overall, the ensemble-averaged MD results indicate that both the WT and mutant complexes retained stable global conformations throughout the simulations (S6 Table in S1 File). The observed differences are more likely associated with localized conformational adjustments and changes in flexibility, particularly near the protein–protein interface. These local changes affect the electrostatic and hydrophobic interactions involved in complex formation and thereby contribute to the observed differences in binding affinity. Overall, the RMSD, RMSF, and Rg analyses suggest that the effects of the substitutions are mainly reflected in local structural dynamics and interfacial interactions.

### 3.3. Mutation-induced perturbations in hydrogen bond networks

To further understand the structural changes induced by mutations, we analyzed the hydrogen-bond network around the mutation sites for R55V, W67V, Y85I, and H86S. Hydrogen bonds play a crucial role in the stability and organization of the structure of proteins, and disruption of these bonds leads to significant changes in protein dynamics [73]. Following the RMSD, RMSF, and Rg results, which suggest increased flexibility and potential destabilization of the MT systems, we expected a similar disruption of hydrogen-bond networks perturbed by the same mutations. For this purpose, the number and the occupancy of the hydrogen bond networks were examined around the residues in the 3.5 Å neighborhood of the mutation sites. Significant difference of number and occupancy of hydrogen bonds were observed in all the studied systems (Fig 10). WT structures revealed up to 15 hydrogen bonds, and many of these had high hydrogen bond occupancy. In general, the mutations resulted in a clear change in the hydrogen-bonding network and the creation of new hydrogen bonds. The hydrogen-bond network changes observed here provide a structural picture of the increased RMSD and RMSF and the reduced structural stability that accompany increased flexibility.

**Fig 10:**
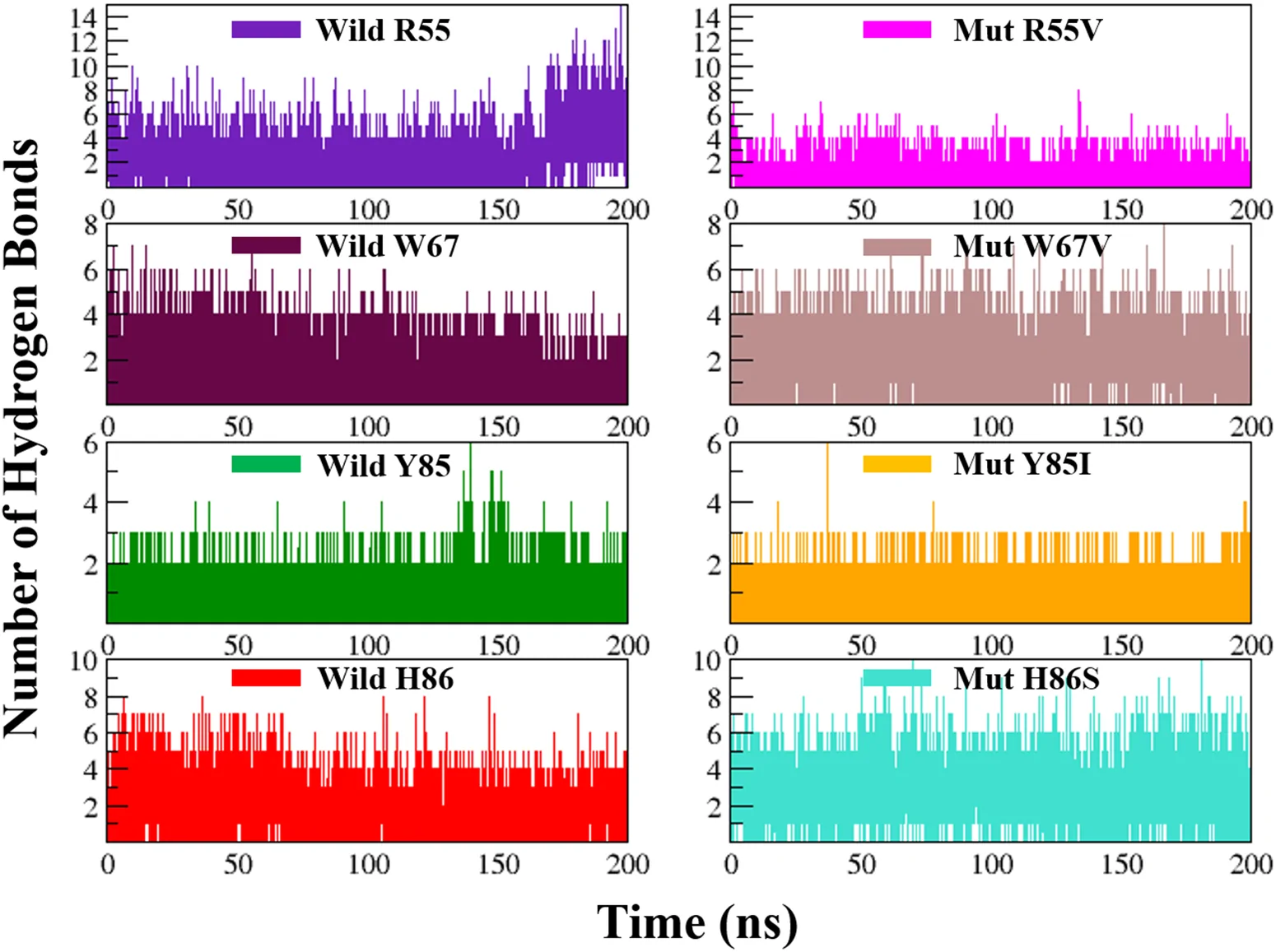
Dynamic trajectory profiles of interfacial hydrogen bond counts. Interfacial hydrogen bonds formed at key mutation positions across WT and mutant systems (residue numbers 55 and 67 in chain B) and (residue numbers 85 and 86 in chain A) with respect to simulation time.

Hydrogen-bond analysis revealed differences in the interfacial hydrogen-bonding patterns among the WT and mutant complexes. The VMD HBonds analysis identified individual donor-acceptor atom pairs using a distance cutoff of 3.5 Å. For residue-level comparison, the occupancies of distinct donor-acceptor pairs involving each residue were summed and are therefore reported as cumulative residue-level hydrogen-bond occupancy. In the R55V variant (chain B), wild-type Arg55 logged 34 distinct donor-acceptor atom-pair interactions over the trajectory, anchored by a primary contact with Glu127 (88.56% occupancy). Meanwhile, Glu57 engaged in a bifurcated network where individual donor-acceptor pairs such as carboxylate oxygen acceptors (OE1 and OE2) yielded a combined residue-level occupancy of 117.16%, reflecting simultaneous multi-dentate bonding rather than a single interaction exceeding 100%. The VAL55 mutation resulted in a decrease of hydrogen bonds to 30, including weakening of the interaction between GLU57 (76.65%) and loss of GLU127. Loss of these interactions, together with the reduced or abolished interactions involving TRP67 and other residues, is in line with increased RMSD and Rg values, along with the weakened or lost interactions involving TRP67 and other residues.

At position 67, hydrogen bond profiling identified 28 distinct donor-acceptor atom-pair interactions involving wild-type Trp67 over the trajectory, anchored by a persistent interaction with Phe113 (81.86% occupancy). In the W67V mutant, 29 unique atom-pair interactions were logged; while the Glu127 contact was lost, a dominant interaction emerged with Asn91 (98.88% occupancy), alongside new contacts with Leu88 and Thr90 that reflect localized dynamic shifts.

There is a reduction in the number of hydrogen bonds from 27(WT) to 24(MT) in association with the Y85I mutation in chain A. The LEU94-associated interaction showed a decrease in occupancy from 79.39% in the WT structure to 59.61% in the mutant. ILE85 formed new interactions with THR204 (0.10%) and HIS217 (0.21%),indicating an observable change in the local interaction environment. The increased RMSD and Rg values correlate with the weakening and redistribution of hydrogen bonding, indicating the possible destabilization of the protein and interaction energies.

The H86S mutant of chain A showed differences in the hydrogen-bonding pattern compared with the WT system. The WT configuration exhibited 31 distinct hydrogen-bonding interactions throughout the trajectory, with notable interactions involving ASP84 (59.24%) and LEU86–GLU127 (49.05%). In contrast, the H86S mutant showed 28 unique interaction pairs alongside marked occupancy redistribution. The mutant system had a SER86 residue that was in key contact (96.06% occupancy) with ASP84, whereas in the WT system, the contact between HIS86 and ASP84 was weak (0.41%).

The mutations led to changes in the hydrogen-bonding networks, including loss of hydrogen bonds and altered bond occupancies (Fig 11). These results agree with the increment in RMSD, RMSF, and Rg, indicating that the mutations affect the structural stability and dynamic properties of IL-17A. Hydrogen bond disruption/reorganization may affect the dynamics of proteins and influence the binding and/or functional characteristics of IL-17A.

**Fig 11:**
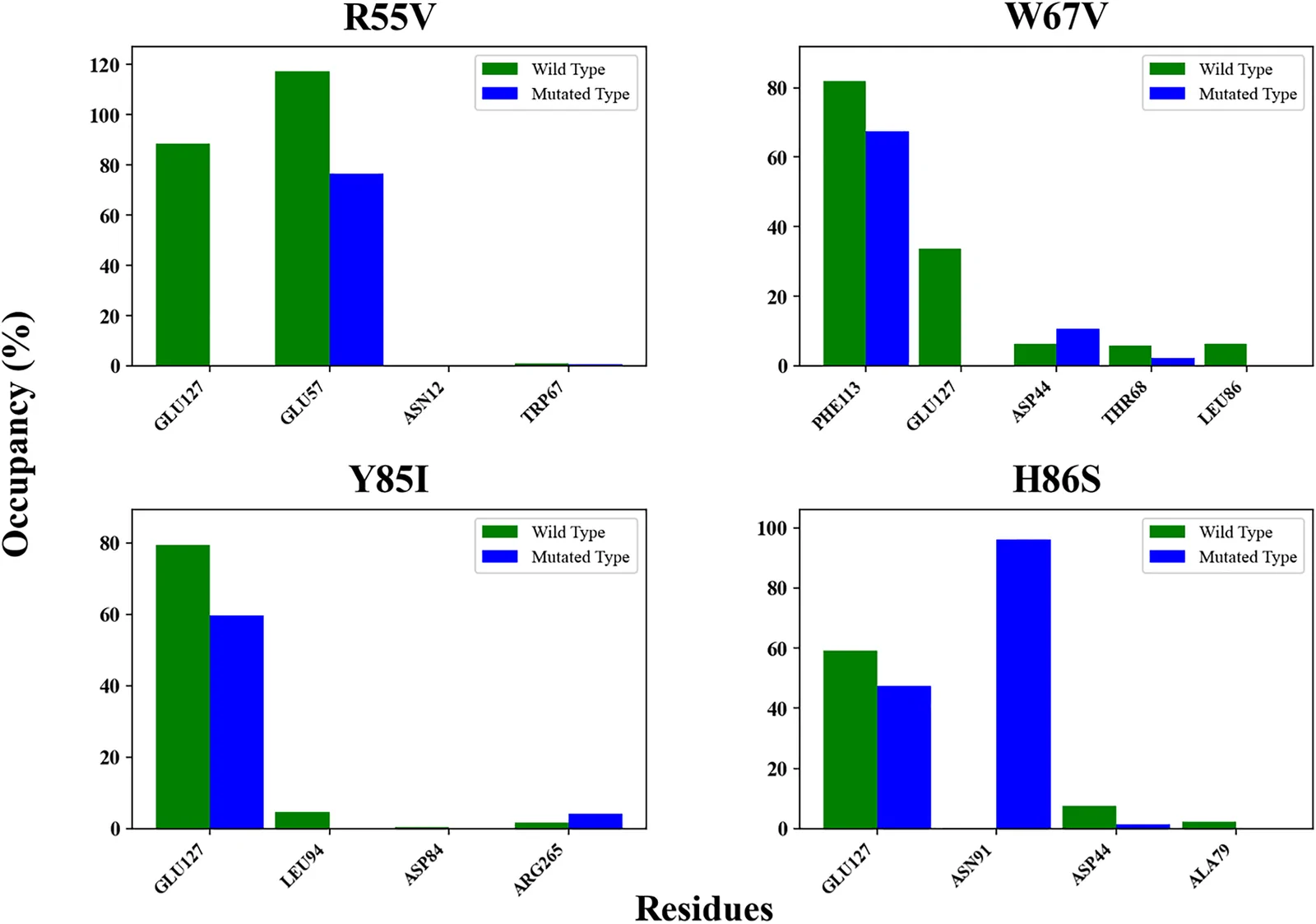
Cumulative residue-level hydrogen bond occupancies. Selected residues of the wild-type and mutant IL-17A are shown by bar charts for R55V, W67V, Y85I, and H86S mutations.

### 3.4. Disruption of inter-residue communication networks by mutations

PSN analysis was used to explore how mutations might impact the structural communication and connectivity within the IL-17A/IL-17RA complex. The outcome shows the residue-residue contacts and provides insights into differences in communication patterns between WT and MT. In this representation, amino acids are nodes and interactions are represented by edges. Highly connected residues are important for the stability and function of proteins because the residues form network communities [74,75]. PSN analysis based on a 100 ns MD trajectory revealed that the structural organization of the IL-17A/IL-17RA complex was quite different between the systems. A well-distributed network, with 489 linked nodes, 575 links, 83 hubs, and 298 hub-mediated links, was observed for the WT system. These findings suggest a strong interaction network, which is probably required for the function of IL-17A/IL-17RA complex.

In contrast, the R55V mutant exhibited a distribution of connectivity and lower participation of hubs, with more linked nodes (495) and links (578), but fewer hubs (78) and hub mediated connections (286). These results suggest that the R55V mutation results in a less stable protein complex, with fewer core interactions, which may impact its functional activities. Likewise, the W67V mutant exhibited higher node connectivity (497) and fewer hubs (71) and hub-mediated links (260), indicating higher dispersion of connectivity architecture. This change suggests the W67V mutant eliminates essential communication networks, disrupting protein stability and interaction. Similarly, the number of hubs (77) and hub-mediated links (277) was also reduced in the Y85I mutant. The loss of hubs also suggests a loss of network cohesion, which could affect its interaction dynamics. The H86S mutant had a moderate level of connectivity, with 72 hubs and 283 hub-mediated interactions, but fewer linked nodes (491) and links (571) than the other mutants (Fig 12). This suggests that the H86S mutant may lead to a less drastic change in the network, which can still impact its overall efficiency, albeit more subtly than the other mutants.

**Fig 12:**
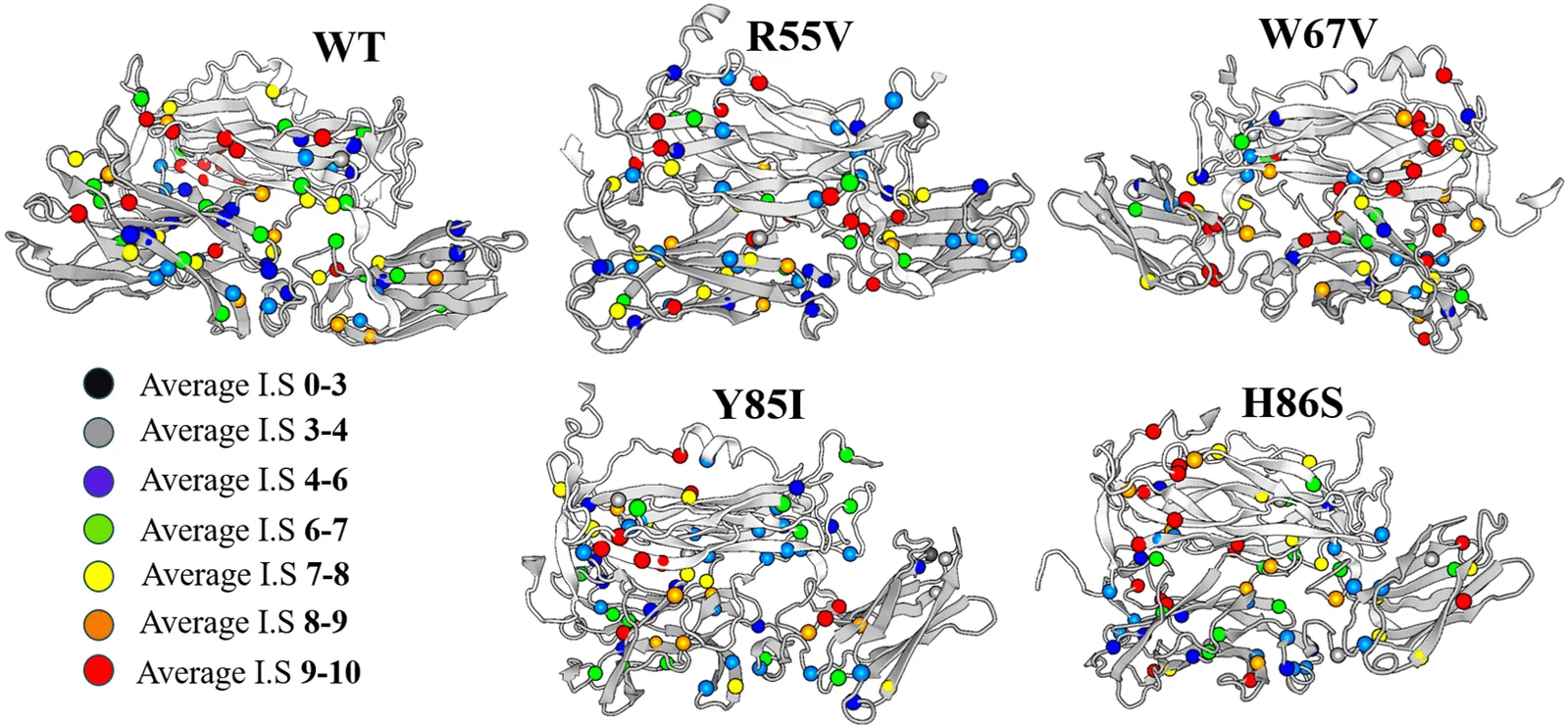
PSN topology and hub distributions. Network analysis at 100 ns showing hubs (circles) in wild-type and mutant IL-17A/IL-17RA complexes, and the interaction strength (I.S).

Further insights were gained from community analysis. The WT had 23 communities, 191 links, and 146 nodes. However, the mutants exhibited a higher number of communities, especially in R55V and Y85I (28 in each case), which indicated a reorganization of network communities (Fig 13, S7 Table in S1 File). The mutant IL-17A systems showed an increase in smaller residue communities, suggesting diminished long-range residue-residue interaction and loss of cooperativity among the interfacial residues that hold the IL-17A dimer together. The breaking up of the interaction network is a signature of a loss of atomic-level cooperativity, resulting in reduced structural stability and dimer integrity. But the number of nodes and links in these communities was reduced in the mutants, suggesting a redistribution or loss of interactions within the network. Therefore, mutations may have disrupted the communication within the critical functional regions of the protein complex.

**Fig 13:**
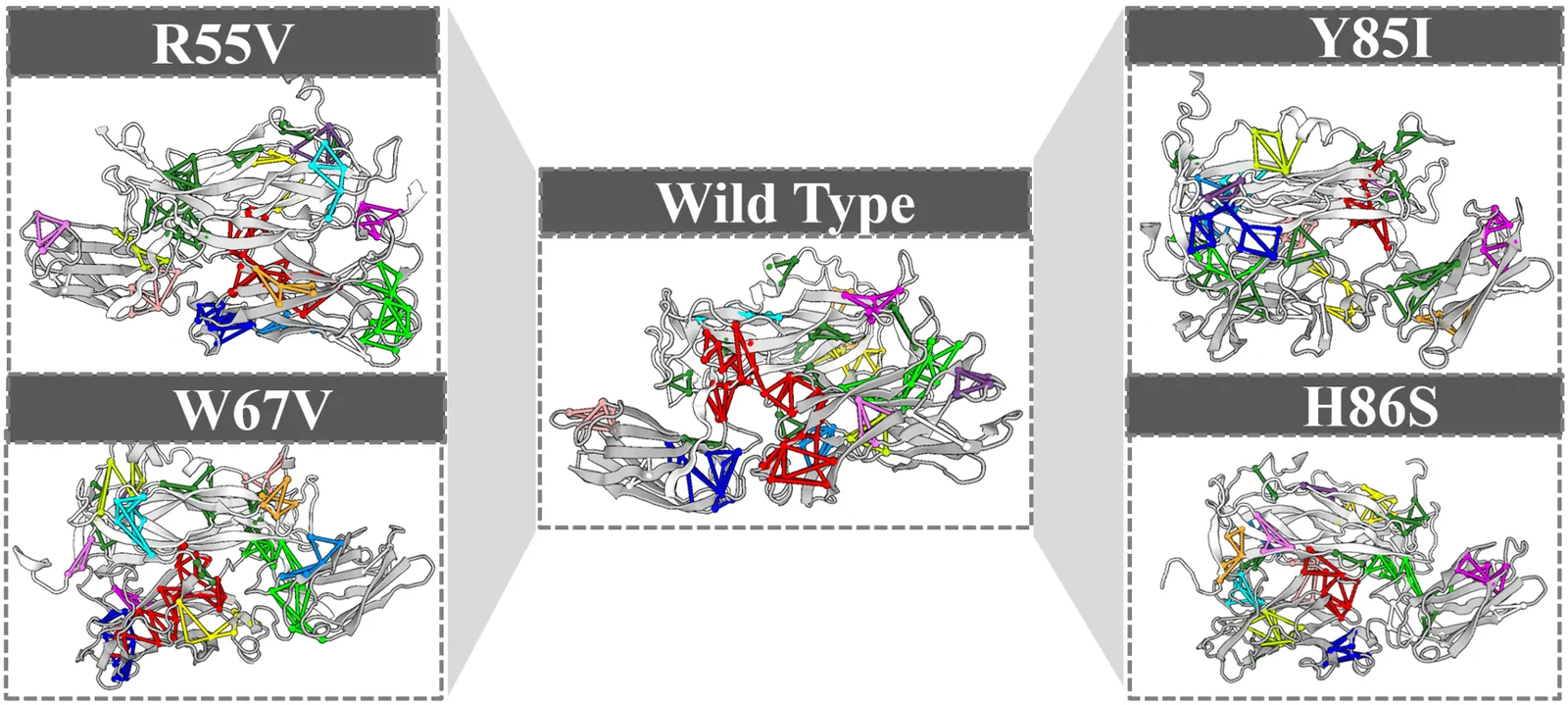
Structural communities in the native and mutant IL-17A/IL-17RA. Structural communities are represented with different colors, with the first community (red) as the largest in terms of nodes, links, and hubs.

Differential PSN analysis showed dramatic changes in the connectivity of the wild-type and variants. The minimum interaction strength (Imin) was greater for the MT (3.24–3.39) than the WT (3.06), possibly due to increased interaction strength to compensate for the loss of interactions. The hub count, on the other hand, decreased in variants (71–78) compared to the WT system (83), suggesting reduced central interactions, which are crucial for protein stability and biological function. Whereas the common hubs count rises, specifically in H86S (59.72%), and specific hubs varied in H86S (40.28%) and R55V (56.41%), indicating alteration in network interaction. The analysis reveals a reduction in hubs and network connectivity, which presumably impairs the protein functionality and stability. The community alignment and hub dispersion are illustrated in Fig 14 and S8 Table in S1 File.

**Fig 14:**
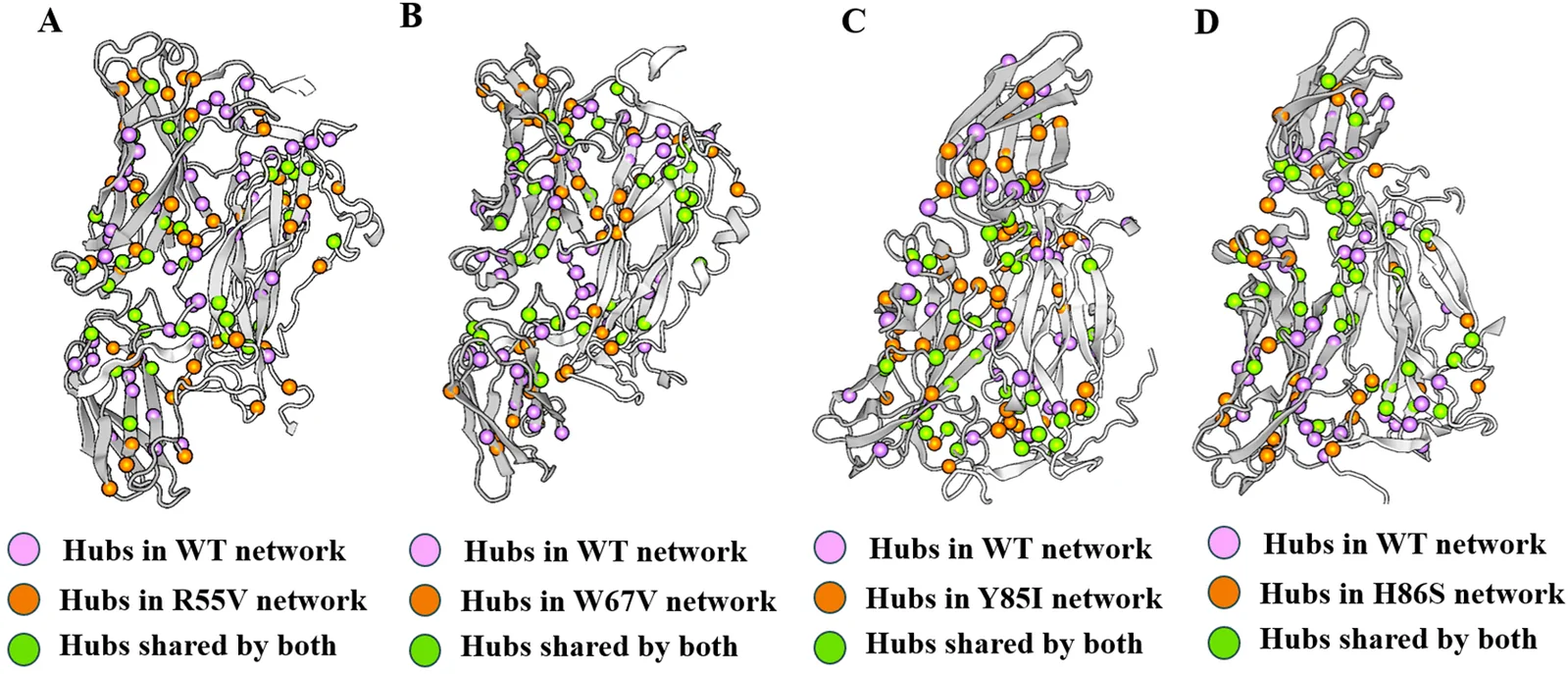
Differential PSN hubs displaying connectivity transitions across WT and mutants: (A) WT vs R55V, (B) WT vs W67V, (C) WT vs Y85I, (D) WT vs H86S.

The consensus PSN analysis (S12 Fig, S9 Table in S1 File) demonstrates the increased connectivity in WT + R55V + W67V network, having additional nodes (516 vs. 514), links (610 vs. 606), and higher hubs (85 vs. 82). Hub-related connections were about similar (311 vs. 310), although previous networks are depicted with increased nodes (143 vs. 136), links (184 vs. 180), and amplified community fragmentation (25 vs. 21 communities). The observations imply that R55V and W67V form more network interactions, while Y85I and H86S showed decreased connectivity, which might affect the protein stability.

### 3.5. Principal Component Analysis (PCA) and Free Energy Landscape (FEL)

Principal Component (PC) analysis is a protocol that decreases data space by mapping atomic motions in terms of principal components, depicting conformational variations, and supporting recognition of principal fluctuations. To gain a holistic perspective of dynamic fluctuations and stability profile, the energy landscape of different conformational states was studied. The critical motions, obtained from PCA, are the essential motions used to describe the protein dynamics [76,77] of IL-17A.

Following backbone alignment and estimation of the PC values, trajectory snapshots from the 50–100 ns fraction of the simulation run were retrieved and cumulative variance illustrates the structural integrity and flexibility. This enabled us to capture representative conformations while minimizing the influence of transient fluctuations. In the WT complex, the conformations are spread out along the PC1 by ±50, and about 50% of the variance along PC1 and 10% along PC2, indicating a clear global free-energy minimum. This indicates the stable protein structure which facilitates its normal functions. As shown in Fig 15, comparative PCA and FEL analysis of both the WT and MT system reveals considerable flexibility and dynamics.

**Fig 15:**
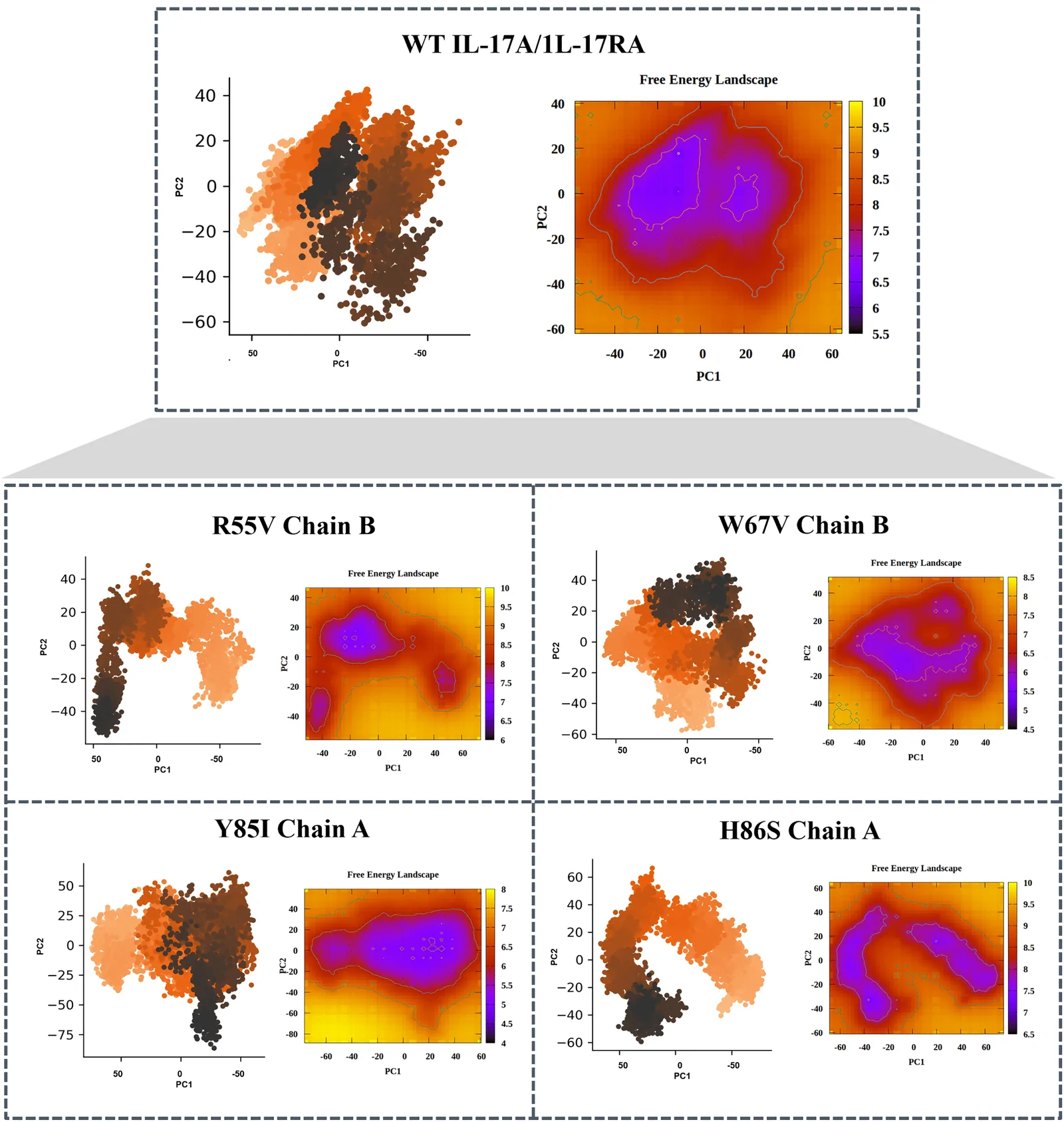
Structural and stability changes of WT and MT systems, showing their conformational dynamics. Contours are plotted as a function of principal components (PC1 and PC2). The energy gradient from purple (low energy) to yellow (high energy) indicates the stability of conformations. The color bar in each panel represents the actual calculated free-energy range of the respective complex, referenced to its most populated state ∆G = 0 kJ/mol.

The large dispersion of PC1 metrices for R55V indicates a more flexible structure with two minima of the FEL (structure transitions) that can be found on the global scale, the energetic landscape is therefore complex. However, W67V showed less flexibility with well-defined FEL global minima, indicating local region flexibility but global stability. While Y85I possesses more flexibility with perturbed FEL and Scattered PCA points, showing destabilization and global fluctuations. This will affect protein function. The most conformationally diverse mutant was H86S, which had scattered PCA points and disrupted FEL, indicating extensive destabilization, which is likely to affect protein stability and function. The WT protein, on the other hand, had a stable energy basin and little conformational change, revealing a stable structure. In summary, the wild-type protein was stable while the mutants were flexible and unstable, showing how mutation can regulate protein conformation, energy landscape, and stability that alter biological function. The representative structures corresponding to the global FEL minima were obtained to describe the most stable conformations in the IL-17A systems (S13 Fig in S1 File).

### 3.6. Structural and dynamic perturbations in IL-17A/IL-17RA complex revealed by correlated motion analysis

To understand the effect of hotspot mutations on the dynamics of the IL-17A/IL-17RA complex, we have used the MD simulation trajectories for the DCCM analysis. This analysis identifies the degree of correlation (positive, red) and anti-correlation (negative, blue) of motions between residues, reflecting the concerted or anti-concerted motion of residues during the simulation. For the WT complex, a pronounced pattern of positive correlations is seen along the diagonal, suggesting a stable concerted distribution of motions in both the IL-17A (Chains A: residues 1−132, and B: residues 133−264) and its interaction domain with IL-17RA (residues 265−527). This is a dynamic network that is conserved for complex functionality. The R55V mutant shows a dramatic decrease in correlated dynamics and an increase in anti-correlated dynamics, mostly between residues 50−200 and 400−450. This suggests inter-domain communication failure across long distances and dysfunction of the allosteric network with repercussions on functional coordination. Similarly, the W67V complex revealed an increase in anti-correlated segments that were found within residues 400−500, corresponding to a reduction in communication of the protein, affecting important dynamics that is key to receptor-ligand stability. Y85I variant demonstrates the dynamic modifications with elevated anti-correlations as well as lower positive correlations in the region 80−120. These localized changes reveal the reduction of structural integrity at a key point of dynamics as it relates to ligand binding or signal transduction. The H86S mutant case exhibited a global loss of correlation and the appearance of anticorrelation between 30−120 residues, indicating that the dynamic structure of the complex has been greatly modified, potentially leading to a change in the flexibility of the complex necessary for an efficient binding and activation of the receptor. To summarize, the current results indicate that mutations at the important hotspot residues of IL-17A have a significant impact on the dynamic correlation networks of the IL-17A/IL-17RA complex. These changes will impact complex integrity and signaling likely as illustrated in Fig 16.

**Fig 16:**
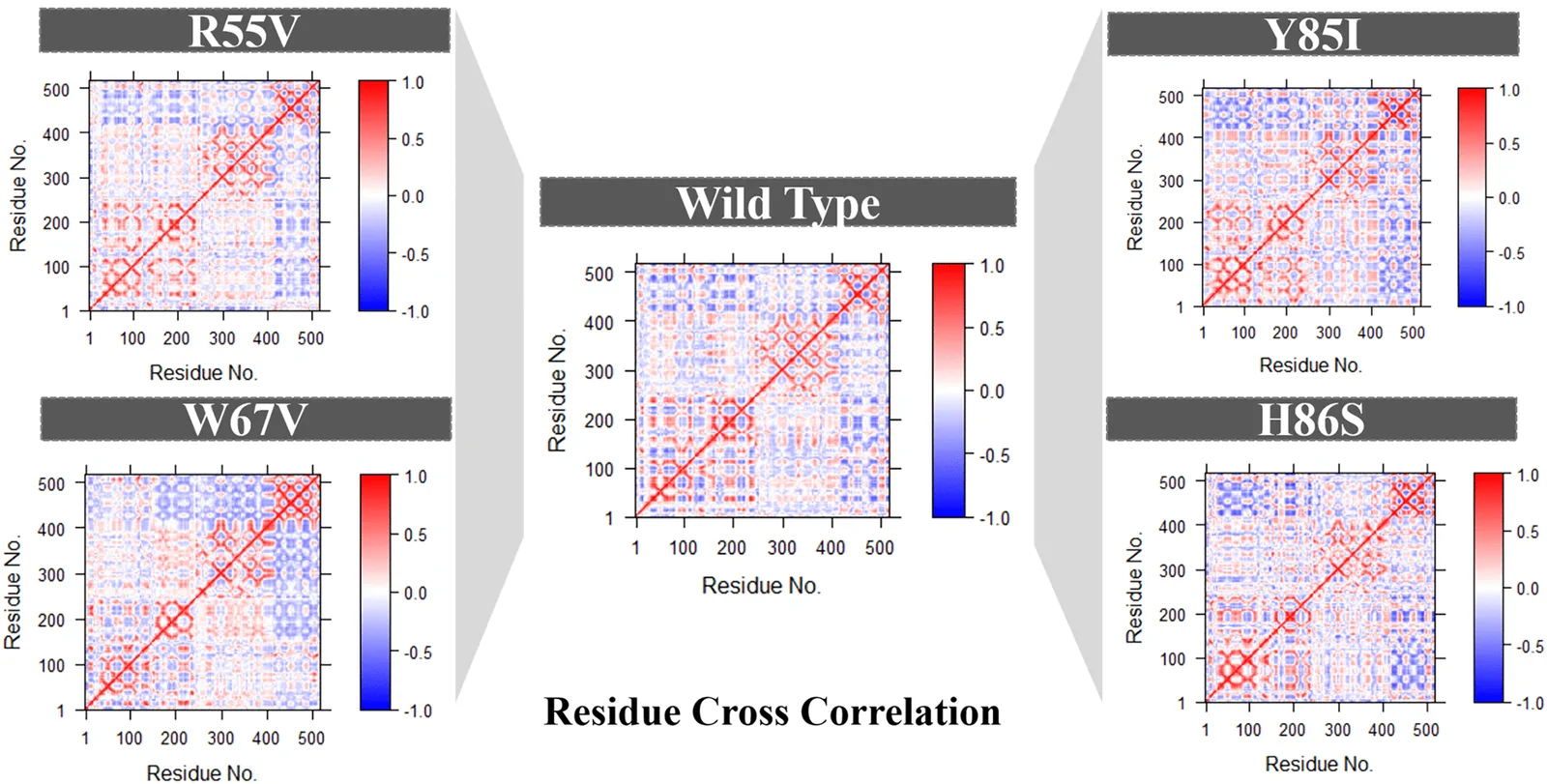
Residue motion correlation analysis of the wild-type and mutant IL-17A/IL-17RA complexes. Cross-correlation heatmaps illustrating correlated (red) and anti-correlated (blue) residue motion to indicate the strength and direction of interactions between residues.

### 3.7. Atomistic structure analysis

The two subdomains D1 and D2 create binding pockets to a large extent governing the interaction between the inflammatory signaling molecule IL-17A and its receptor, IL-17RA. These pockets are rich in a network of hydrophobic interactions, electrostatic interactions, and hydrogen bonds between key residues of IL-17A (Chain A and B) and IL-17RA (Chain C) within a 3.5 Å distance, which demonstrates the complexity of the binding.

The D1 subdomain serves as a critical interface. Notably, IL-17A residues Leu53, Arg55, Trp67, and Ile65 engage with Glu127, His131, and Ser146 on IL-17RA. Leu53 engages in hydrophobic interactions, while Arg55 is crucial for electrostatic and hydrogen bonding. Its guanidinium group forms key hydrogen bonds with Glu127 (2.28 Å, 1.69 Å) and Ser146 (3.38 Å), anchoring the complex (Fig 17). The mutation to valine (MT V55) destroys these interactions, removing essential electrostatic interactions and compromising the binding. Likewise, Trp67 directly stabilises the interface by forming hydrophobic and π-π interactions, and hydrogen bonds with Glu127 (3.15 Å and 3.14 Å). Substitution with valine (MT V67) eliminates all these interactions because of the absence of the aromatic side chain and weakens the interaction, despite potential weak hydrogen bonding with the backbone.

**Fig 17:**
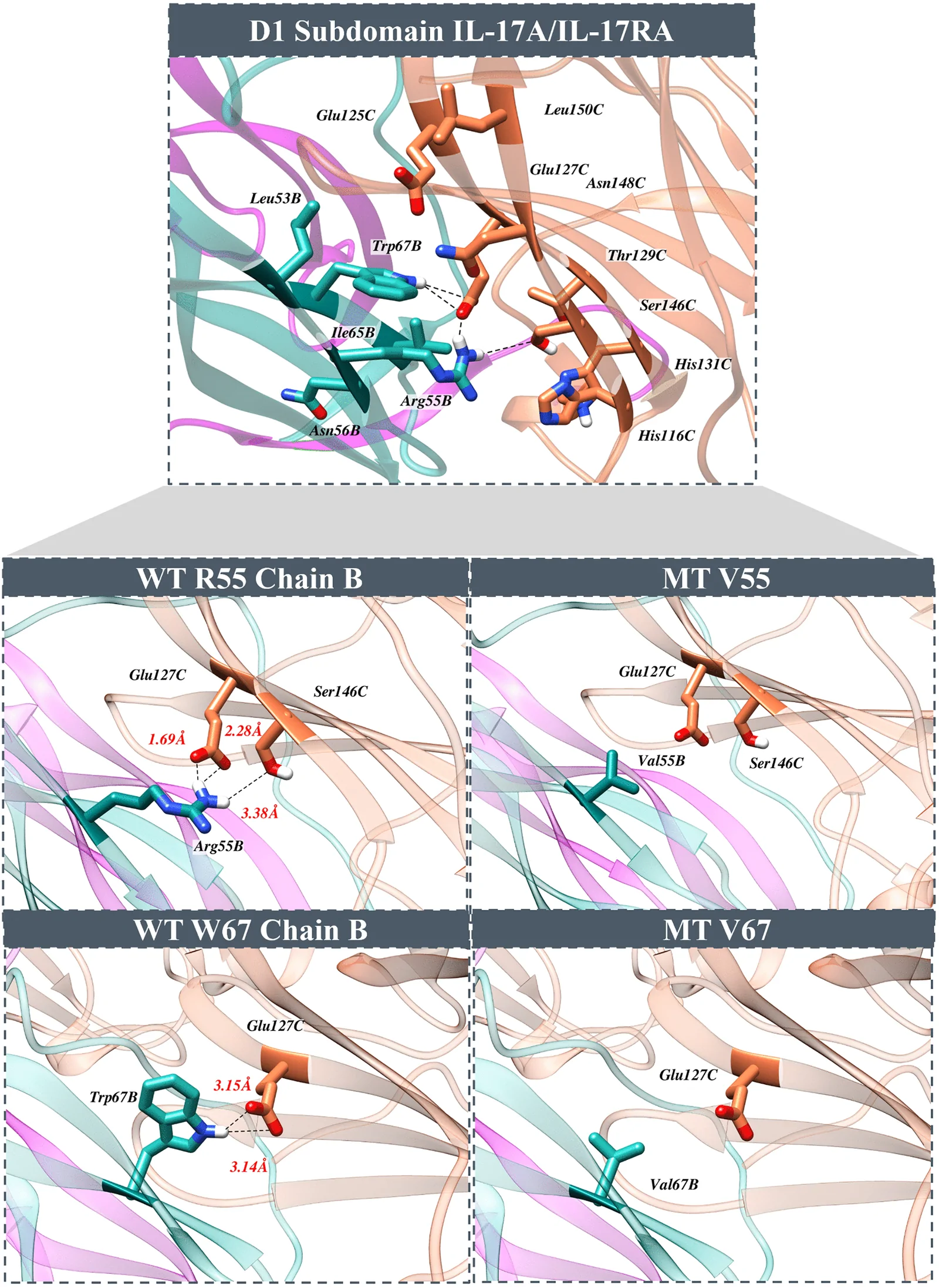
Interactions in the D1 subdomain pocket of wild-type and mutant complexes. The top left panel illustrates wild-type R55 interactions, while the bottom left is the R55V mutation. The top-right panel is for wild-type W67, and the bottom-right for the W67V mutation.

Therefore, it is proposed that Arg55 and Trp67 are critical residues in IL-17A for binding to IL-17RA. Arg55 is critical for strong electrostatic and hydrogen bonding networks, while Trp67 provides hydrophobic, π-π, and hydrogen bonding interactions. Substituting these with valine eliminates these interactions, destabilizing the complex and causing a decrease in binding affinity, underscoring the role of amino acid side chain properties in protein-protein interactions.

The D2 subdomain of the IL-17A/IL-17RA complex has a critical binding site that is maintained by hydrophobic, electrostatic, and hydrogen bond interactions between IL-17A (Chain A) and IL-17RA (Chain C). IL-17A residues Tyr85, His86, Asn88, and His129 and IL-17RA residues Arg265, Gln200, Met218, Glu216, and Thr204 are critical for this interaction. IL-17A residue Tyr85 makes a key hydrogen bond (3.48 Å) to the carbonyl oxygen of Gln200 in IL-17RA, as illustrated in Fig 18. This linkage effects considerably to complex stability. This interaction is prevented by substituting the tyrosine with isoleucine (MT I85), without the hydrogen bonding interaction, and reduces the strength of the local region. His86 also contributes to the interaction by interacting with Glu216 (3.15 Å) and Thr204 (3.94 Å) in IL-17RA. The imidazole group of His86 can hydrogen bond and electrostatically interact. This His86 to serine (MT S86) replacement affects the interactions as a result of a smaller side chain and reduced hydrogen bonding potential which exposes weaker contacts and interface perturbation. To summarise, the residues Tyr85 and His86 are important for stabilising the D2 subdomain through specific hydrogen bonding and electrostatic interactions with IL-17RA. The mutations to Ile85 and Ser86 alter these interactions, reducing the binding strength and weakening the stability of the complex, as revealed by structural and molecular dynamics studies.

**Fig 18:**
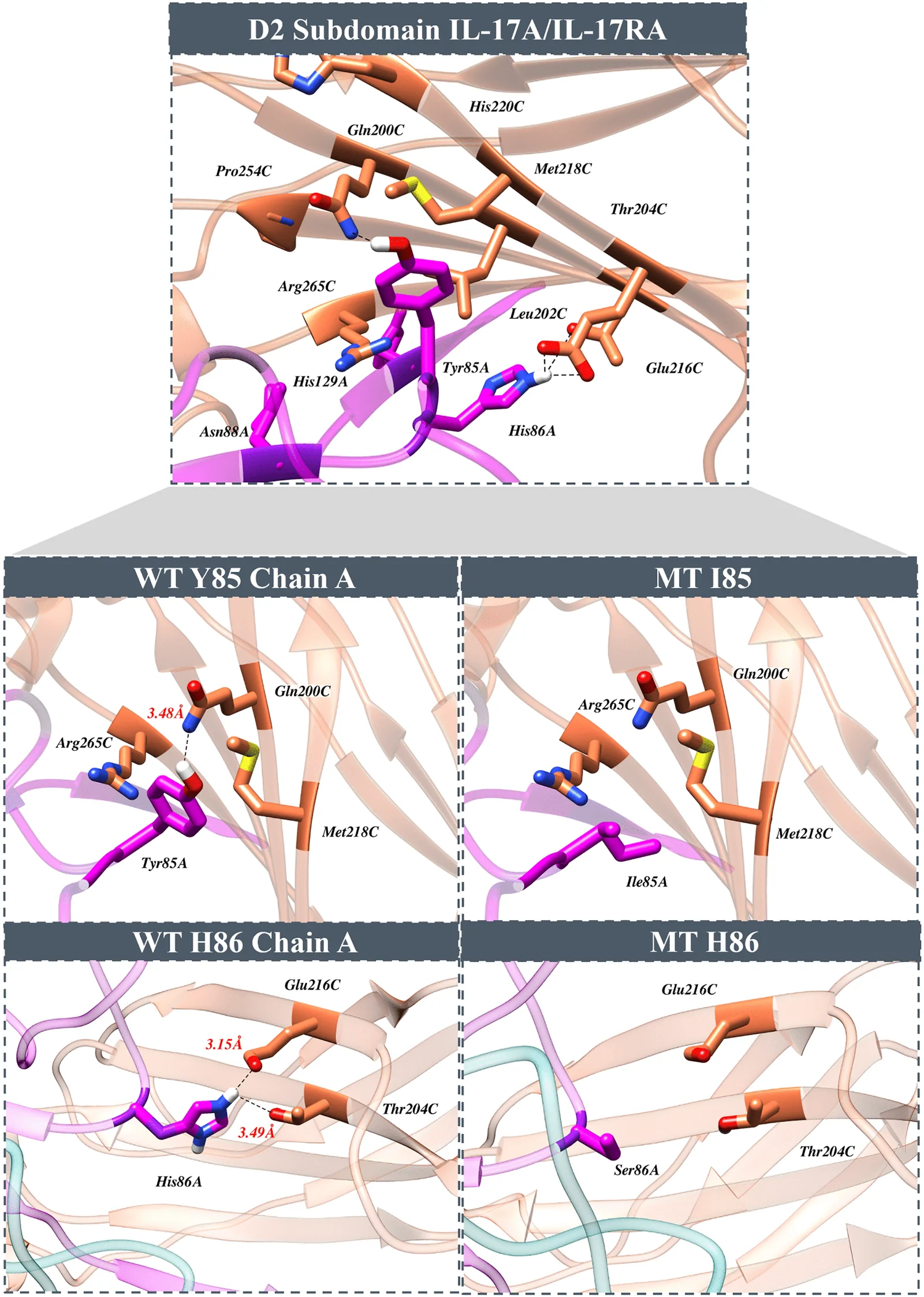
Interactions at the D2 subdomain pocket in WT and MT complexes. The top-left panel shows interactions of wild-type Y85, bottom-left is the Y85I mutation. The top-right shows the interactions of wild-type H86, and the bottom-right the H86S mutation.

### 3.8. MM-GBSA calculation

To assess the effect of the mutations on the binding free energy of the IL-17A-IL-17RA complex, we used the Molecular Mechanics Generalized Born Surface Area (MM-GBSA) method. MM-GBSA is a widely used and computationally efficient method for binding free energy calculations and assessing the stability of protein-ligand and protein-protein interactions [78]. The binding free energies (ΔG_MMGBSA_) of the WT and the MT system were calculated by a component-resolved calculation. This requires calculating each in their main energy contributions: Van der Waals (ΔE_VDW_), electrostatic energy (Δ_EELE_), polar solvation energy (ΔG_GB_), and the estimated non-polar solvation energy by solvent-accessible surface area (∆G_SA_). Energy decomposition, on the other hand, gives insight into the energy contribution that facilitates the protein-protein interaction.

To account for conformational fluctuations across the 200 ns MD trajectories, we evaluated binding free energies using representative snapshots extracted at 0, 50, 100, 150, and 200 ns, capturing the dynamic relaxation toward the equilibrium state. Table 2 reports values as the mean ± standard deviation, with detailed individual energy components in S10 Table in S1 File. At the Chain B–Chain C interface, the WT complex exhibited the most favorable binding free energy, with an initial value of −114.31 kcal/mol, compared with −103.49 kcal/mol for R55V and −62.11 kcal/mol for W67V. At 200 ns, the corresponding values were −97.41, −89.13, and −85.01 kcal/mol, respectively. Consistent with these observations, the ensemble-averaged values were −106.47 ± 8.83 kcal/mol (WT), −97.62 ± 5.24 kcal/mol (R55V), and −80.68 ± 18.72 kcal/mol (W67V). These results indicate that both substitutions weakened the predicted binding affinity, with W67V producing the largest energetic perturbation and the greatest variability. A similar trend was observed at the Chain A–Chain C interface, where the initial binding energies were −92.58 kcal/mol (WT), −80.66 kcal/mol (Y85I), and −70.66 kcal/mol (H86S). At 200 ns, these values were −86.84, −82.60, and −79.51 kcal/mol, respectively. The corresponding ensemble averages were −92.55 ± 11.29 kcal/mol, −82.03 ± 5.62 kcal/mol, and −80.82 ± 7.23 kcal/mol for WT, Y85I, and H86S, respectively, demonstrating reduced binding favorability following both substitutions.

**Table 2:** Ensemble-averaged MM/GBSA binding free energy components. Binding free energies (kcal/mol) of the WT and MT systems of the IL-17A/IL-17RA complex calculated from representative structures sampled at 0, 50, 100, 150, and 200 ns of the MD trajectories. Values are presented as mean ± standard deviation.

Systems	∆E_VDW_	∆E_ELE_	∆G_GB_	∆G_SA_	∆G_MMGBSA_
**WT (Chain B–Chain C)**	−127.17 ± 8.38	−463.08 ± 84.75	501.29 ± 71.21	−17.51 ± 0.82	**−106.47 ± 8.83**
**R55V**	−121.01 ± 12.22	−313.95 ± 69.62	353.92 ± 53.28	−16.58 ± 1.29	**−97.62 ± 5.24**
**W67V**	−112.13 ± 15.71	−379.66 ± 135.58	426.51 ± 131.82	−15.41 ± 2.12	**−80.68 ± 18.72**
**WT (Chain A–Chain C)**	−119.26 ± 5.44	−133.59 ± 61.55	175.89 ± 53.21	−15.60 ± 0.71	**−92.55 ± 11.29**
**Y85I**	−113.47 ± 7.36	−165.77 ± 59.99	211.77 ± 60.53	−14.56 ± 0.95	**−82.03 ± 5.62**
**H86S**	−114.03 ± 11.22	−129.03 ± 53.17	176.67 ± 53.52	−14.43 ± 0.88	**−80.82 ± 7.23**

A clear inverse correlation was observed between the stability of the mutant systems and their calculated binding free energies, as increased destabilization of the systems corresponded to less negative (higher) binding free energies, suggesting weaker interactions. Energy component analysis revealed that mutation-induced drops in binding affinity were driven primarily by ∆E_VDW_ and ∆E_ELE_ interactions, whereas polar ∆G_GB_ and non-polar ∆G_SA_ solvation terms remained largely comparable. This indicates that the mutations mainly affect the short-range non-polar interactions and the long-range electrostatic complementarity at the binding interface. In summary, the binding free energy calculations undeniably establish the fact that the mutations studied substantially reduce the binding free energy of the IL-17A-IL-17RA complex, confirming the qualitative inference from the atomistic analysis. Residue-based energy profiling (Fig 19 and S11 Table in S1 File) also suggests that interfacial pocket residues contribute overall binding energy, with the mutant systems showing substantial changes in energy profiles relative to the wild type, emphasizing the energetic role of these residues in maintaining the stability of the IL-17A/17RA complex.

**Fig 19:**
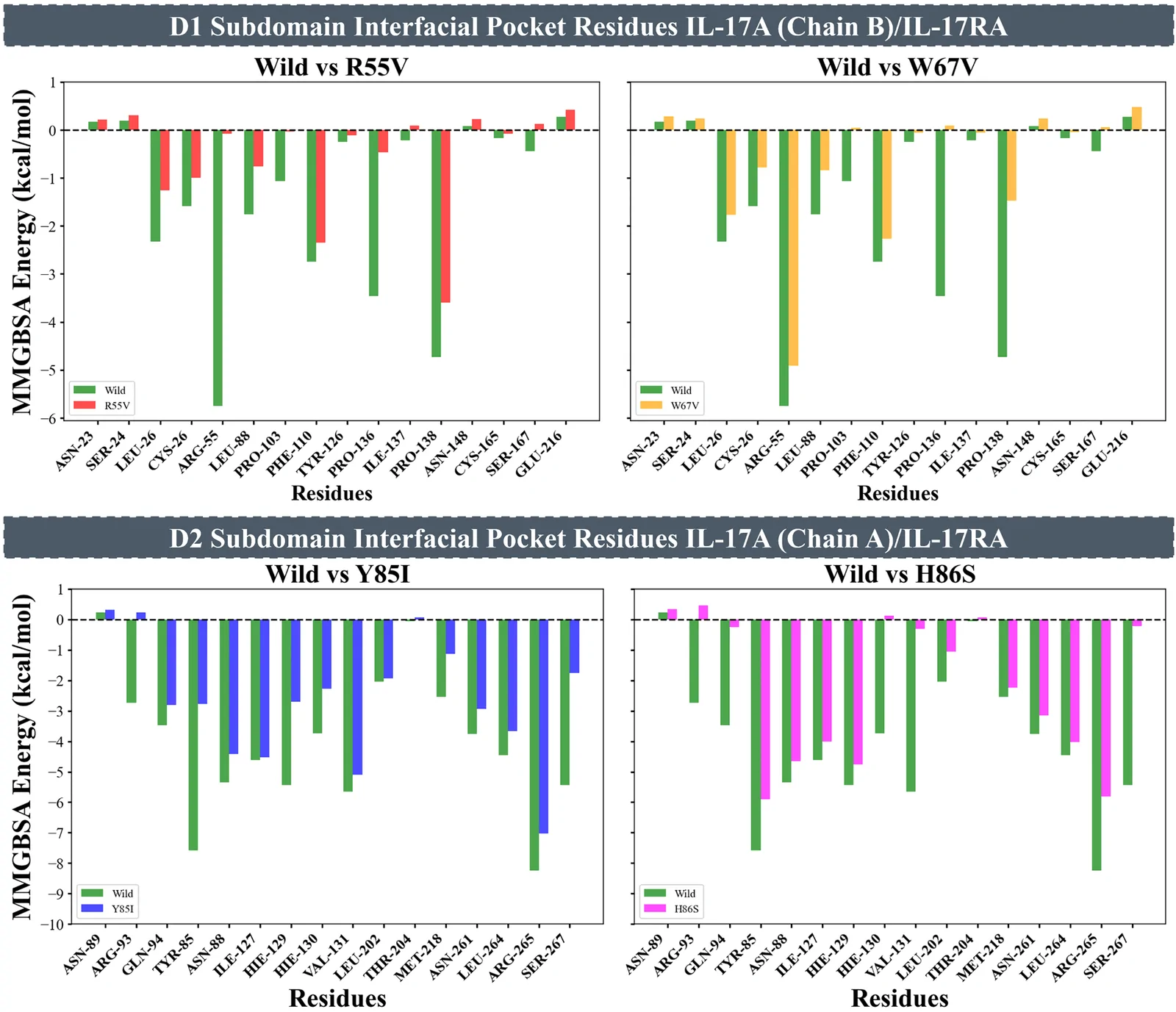
Per-residue MM/GBSA free energy decomposition. Contributions of binding free energy of the interface pocket residues of IL-17A binding to IL-17RA.

Beyond elucidating the energetic consequences of the mutations, the MM/GBSA binding free-energy and per-residue decomposition analyses provide a rational blueprint for future structure-based drug design against IL-17A. The observed reductions in electrostatic and van der Waals contributions in the mutant systems highlight the importance of specific interfacial regions in maintaining IL-17A/IL-17RA recognition. In particular, R55, W67, Y85, and H86 represent important energetic hotspots that may serve as structural anchors for designing small molecules or peptidomimetics targeting the receptor-binding interface. Ligand optimization around these regions could be explored to promote favorable electrostatic and van der Waals interactions and thereby improve binding to the targeted interface. Furthermore, the distinct energetic contributions of the chain-specific hotspots provide a basis for exploring ligand selectivity toward IL-17A over closely related cytokines such as IL-17F. Our structural dynamics and energetics results provide an integrated mechanistic picture of the effect of the mutations on IL-17A. Mutations were designed at the defined contact residues that are centralised in the structure (R55V, W67V, Y85I, and H86S) and were found to disrupt the stability of the IL-17A/IL-17RA complex by disrupting key hydrogen bonds and reducing the van der Waals interactions that strongly contribute to the cytokine/receptor interaction. The loss of interfacial interaction is manifested by a decrease in the count of hydrogen bonds, a decrease in the binding energy and an increase in local flexibility of the MT systems. Overall, it is expected that this structural disruption will impair the ability of IL-17A to bind productively to the receptor and thereby prevent receptor activation and the following downstream pro-inflammatory signaling pathway. The results provide, as a whole, a comprehensive understanding of the role of the variants on the structure and function of IL-17A.

## 4. Conclusion

Because of the time consuming, laborious, and resource-intensive nature of traditional in vitro methods, the effect of four mutations (R55V, W67V, Y85I and H86S) on the structural integrity of the IL-17A/IL-17RA complex was explored using a computational model. In silico mutagenesis of key residues in IL-17A (R55V and W67V in chain B; Y85I and H86S in chain A) revealed distinct localized structural perturbations at the protein-protein interface across independent simulation replicates. This study provides critical atomic-level insights into the molecular determinants governing IL-17A/IL-17RA complex assembly. More importantly, substitution of the IL-17A functional residues with the corresponding residues of IL-17F substantially compromised interface stability, uncovering specific residues required for high-affinity receptor binding. Analysis of protein stability of the mutant systems compared to the wild-type, primarily driven by the loss of critical hydrogen bonds and hydrophobic interactions, which potentially affected interfacial stability. Network analysis also demonstrated considerable loss of connectivity and functionality, particularly for W67V and Y85I, suggesting alterations in inter-subunit communication. Finally, conformational flexibility and energy profiles demonstrated that the point mutations induce localized flexibility and conformational-state distribution across the mutant systems, compared with the WT complex maintaining an optimal thermodynamic balance between stability and flexibility. These computational insights complement the experimental data by clarifying how these point mutations affect receptor binding and may subsequently affect downstream signaling and associated mechanisms. Overall, these findings provide a sound computational baseline for designing targeted therapeutic drugs for the modulation of IL-17A/IL-17RA interactions that may be used to treat inflammatory diseases, and provide valuable insights for future studies on the biology of macromolecular interactions.

## Supporting information

S1 File The supporting information file contains Supporting Figures S1-S13 and Supporting Tables S1-S11, providing additional figures, data, analyses, and detailed results that complement and support the findings presented in the main manuscript. (DOCX)
