# Supplementary material for: Mutation-induced structural and dynamic perturbations at the IL-17A–IL-17RA interface: Therapeutic insights from multi-replicate molecular dynamics simulations
Source: PLoS One. 2026 Nov 6;21(11):e0361876. doi: 10.1371/journal.pone.0361876 (PMC13742950; doi:10.1371/journal.pone.0361876)
Supplement: Supplementary file 1 [file pone.0361876.s001.docx]

***Mutation-Induced Structural and Dynamic Perturbations at the IL-17A–IL-17RA Interface: Therapeutic Insights from Multi-Replicate Molecular Dynamics Simulations***

Afsheen Razzaq^a^, Madiha Sardar^a^, Mamona Mushtaq^b^, Mohammad Nur-e-Alam^c^, Muhammad Nabeel Ghayur^d^, and Zaheer Ul-Haq^*,b^

^a^ H. E. J. Research Institute of Chemistry, International Center for Chemical and Biological Sciences, University of Karachi, Karachi-75270, Pakistan

^b^Dr. Panjwani Center for Molecular Medicine and Drug Research, International Center for Chemical and Biological Sciences, University of Karachi, Karachi-75270, Pakistan

^c^ Department of Pharmacognosy, College of Pharmacy, King Saud University, P.O. Box. 2457, Riyadh 11451, Kingdom of Saudi Arabia

^d^ Kentucky College of Osteopathic Medicine, University of Pikeville, Pikeville, KY, USA

**Corresponding author:**

Zaheer Ul-Haq; [zaheer.qasmi@iccs.edu](mailto:zaheer.qasmi@iccs.edu)

**S1 Fig. Sequence alignment of human IL-17A and IL-17F.** Highlights conserved regions, sequence variations, and key receptor-interacting hotspot residues. Identical residues are marked with an asterisk (*), and conserved residues with a colon (:).

**S2 Fig. Interfacial interaction network maps generated from PDBsum.** Interaction diagrams of the IL-17A/IL-17RA wild-type and mutant systems R55V, W67V (mutations in chain B), and Y85I, H86S (mutations in chain A) highlight the impact of mutations on the interaction network between IL-17A and receptor IL-17RA. Orange represents hydrophobic interactions, while hydrogen bonds are indicated in blue.

**S3 Fig. Backbone RMSD Trajectories (Replica 2).** Wild-Type (black) and Mutant IL-17A (R55V, red; W67V, green; Y85I, magenta; H86S, orange) over 200 ns from replicate 2 MD simulations, indicating structural deviations.

**S4 Fig. Backbone RMSD Trajectories (Replica 3).** Wild-type and mutant IL-17A–IL-17RA complexes RMSD shown over 200 ns from replicate 3 MD simulations.

**S5 Fig. Multi-replica RMSD trajectory overlay.** RMSD profiles of IL-17A for the wild-type and mutant systems (R55V, W67V, Y85I, and H86S) over 200 ns MD simulations. Three independent replicates are shown: R1 (black), R2 (red), and R3 (green).

**S6 Fig. Per-Residue RMSF Profiles (Replica 2).** RMSF of Wild-Type (black) and Mutant IL-17A (R55V, red; W67V, green; Y85I, magenta; H86S, orange) from replicate MD simulations, showing residue-level flexibility.

**S7 Fig. Per-Residue RMSF Profiles (Replica 3).** RMSF of the wild-type and mutant IL-17A–IL-17RA complexes over 200 ns from triplicate molecular dynamics simulations.

**S8 Fig. Multi-replica RMSF profile overlay.** RMSF profiles of IL-17A for the wild-type and mutant systems (R55V, W67V, Y85I, and H86S) over 200 ns MD simulations, showing a comparison of three independent replicates: R1 (black), R2 (red), and R3 (green).

**S9 Fig. Radius of gyration trajectories (Replica 2).** Rg of Wild-Type (black) and Mutant IL-17A (R55V, red; W67V, green; Y85I, magenta; H86S, orange) during 200 ns replicate simulations, reflecting compactness.

**S10 Fig. Radius of gyration trajectories (Replica 3).** Rg of the wild-type and mutant IL-17A–IL-17RA complexes over 200 ns from triplicate molecular dynamics simulations.

**S11 Fig. Multi-Replica Radius of gyration trajectories overlay.** Comparative Rg profile of wild-type and mutant IL-17A (R55V, W67V, Y85I, and H86S) over 200 ns MD simulations, showing consistency across three independent replicates: R1 (black), R2 (red), and R3 (green).

**S12 Fig.** **Consensus Protein Structure Network (PSN) topology.** Consensus structure network analysis at the 100 ns frame comparing wild type with R55V and W67V mutations (left) to wild type with Y85I and H86S mutations (right). Hubs are represented on the left side, while community structures are shown on the right, highlighting differences in connectivity, hub strength, and community distribution for each system. Interaction strength (I.S.) is indicated within the hubs.

**S13 Fig. Representative Low-Energy Conformations.** Free energy landscape (FEL) plots of wild-type and mutant IL-17A systems, along with their corresponding representative conformations extracted from the global energy minima.

**S1 Table. Impact of mutations on protein stability.** Predictions with ΔΔG values indicating the effect on structural integrity, categorized by stabilizing and destabilizing mutations across Chain A and Chain B using CUPSAT, DUET, DynaMut, and SAAMBE.

| S.NO | Mutants | CUPSAT (ΔΔG) | DUET (ΔΔG) | DynaMut (ΔΔG) | ****SAMBEE**** (ΔΔG) |
| --- | --- | --- | --- | --- | --- |
| Chain A | | | | | |
| 1 | **R55V** | **-1.68** (*Destabilizing*) | **0.237** (*Stabilizing*) | **0.13** (Stabilizing) | **0.57** (*Destabilizing*) |
| 2 | **W67V** | **1.46** (*Stabilizing*) | **-1.214** (*Destabilizing*) | **-1.46** (Destabilizing) | **0.23** (*Destabilizing*) |
| 3 | **Y85I** | **-1.26** (*Destabilizing*) | **-0.908** (*Destabilizing*) | **-1.09** (Destabilizing) | **1.51** (*Destabilizing*) |
| 4 | **H86S** | **-2.58** (*Destabilizing*) | **0.525** (*Stabilizing*) | **1.75** (Stabilizing) | **0.01** (*Destabilizing*) |
| Chain B | | | | | |
| 1 | **R55V** | **-0.67** (*Destabilizing*) | **-0.019** (*Destabilizing*) | **-0.36** (Destabilizing) | **0.85** (*Destabilizing*) |
| 2 | **W67V** | **0.63** (*Stabilizing*) | **-2.491** (*Destabilizing*) | **-2.78** (Destabilizing) | **1.34** (*Destabilizing*) |
| 3 | **Y85I** | **6.48** (*Stabilizing*) | **-0.351** (*Destabilizing*) | **0.06** (Stabilizing) | **1.09** (*Destabilizing*) |
| 4 | **H86S** | **6.02** (*Stabilizing*) | **0.384** (*Stabilizing*) | **1.79** (Stabilizing) | **-0.14** (*Stabilizing*) |

**S2 Table. Changes in binding affinity of the protein induced by mutations.** Structure-based binding affinity changes with ΔΔG values illustrating the effect on protein-protein interactions computed via DDMut_PPI, MCSM-PPI2, BeAtMuSiC, and MOE.

| S.NO | Mutants | DDMut_PPI *ΔΔGAffinity kcal/mol* | MCSM-PPI2 *ΔΔGAffinity* | BeAtMuSiC *ΔΔGAffinity* | MOE |
| --- | --- | --- | --- | --- | --- |
| Chain A | | | | | |
| 1 | **R55V** | **-0.097** (Decreasing affinity) | **0.144** (Increasing affinity) | ***0.01*** *(Decreasing affinity)* | **-0.058** (Increasing affinity) |
| 2 | **W67V** | **-0.028** (Decreasing affinity) | **-0.109** (Decreasing affinity) | ***0.23*** *(Decreasing affinity)* | **-0.087** (Increasing affinity) |
| 3 | **Y85I** | **-0.281** (Decreasing affinity) | **-0.665** (Decreasing affinity) | ***1.06*** *(Decreasing affinity)* | **0.268** Decreasing affinity) |
| 4 | **H86S** | **-0.162** (Decreasing affinity) | **0.340** (Increasing affinity) | ***1.11*** *(Decreasing affinity)* | **0.478** Decreasing affinity) |
| Chain B | | | | | |
| *1* | ***R55V*** | **-0.527** (Decreasing affinity) | **0.233** (Increasing affinity) | ***-0.10 (Increasing Affinity)*** | **0.418** Decreasing affinity) |
| *2* | ***W67V*** | **-1.146** (Decreasing affinity) | **-2.044** (Decreasing affinity) | ***2.68*** *(Decreasing affinity)* | **1.366** Decreasing affinity) |
| *3* | ***Y85I*** | **-0.069** (Decreasing affinity) | **-0.187** (Decreasing affinity) | ***-0.01 (Increasing Affinity)*** | **-0.052** Decreasing affinity) |
| *4* | ***H86S*** | **-0.074** (Decreasing affinity) | **-0.133** (Decreasing affinity) | ***-0.10 (Increasing Affinity)*** | **-0.014** Decreasing affinity) |

**S3 Table. Mean RMSF values for WT and variant systems.** Residue-level fluctuation averages (mean ± SD in nm) for WT and variant systems computed across three independent MD simulation replicas.

| **System** | **Replicate 1 (nm)** | **Replicate 2 (nm)** | **Replicate 3 (nm)** |
| --- | --- | --- | --- |
| **WT** | 0.264764 ± 0.0281916 | 0.250306 ± 0.0568235 | 0.262243 ± 0.020077 |
| **R55V** | 0.299712 ± 0.0314314 | 0.283891 ± 0.0315322 | 0.340237 ± 0.0483839 |
| **W67V** | 0.2684898 ± 0.0295166 | 0.279407 ± 0.0405945 | 0.253985 ± 0.0316581 |
| **Y85I** | 0.306067 ± 0.0511885 | 0.270174 ± 0.0449804 | 0.272522 ± 0.0301257 |
| **H86S** | 0.293303 ± 0.0344352 | 0.275545 ± 0.0397103 | 0.270652 ± 0.028528 |

**S4 Table. Mean RMSF values ( nm± SD ) for WT and variant systems computed across three independent MD simulation replicas.**

| **System** | **Replicate 1 (nm)** | **Replicate 2 (nm)** | **Replicate 3 (nm)** |
| --- | --- | --- | --- |
| **WT** | 0.138166 ± 0.104795 | 0.142099 ± 0.106021 | 0.160074 ± 0.0880742 |
| **R55V** | 0.146252 ± 0.117883 | 0.163121 ± 0.125135 | 0.192952 ± 0.116845 |
| **W67V** | 0.148239 ± 0.106266 | 0.136157 ± 0.118233 | 0.162979 ± 0.103805 |
| **Y85I** | 0.148207 ± 0.116097 | 0.145995 ± 0.0972473 | 0.162346 ± 0.112015 |
| **H86S** | 0.146269 ± 0.111164 | 0.147958 ± 0.136417 | 0.169782 ± 0.111917 |

**S5 Table. Time-averaged radius of gyration (**$\boldsymbol{R}_{\boldsymbol{g}}$**).** Structural compactness values (mean ± SD in nm) computed from three independent 200 ns simulation replicas for WT and mutant complexes.

| **System** | **Replicate 1 (nm)** | **Replicate 2 (nm)** | **Replicate 3 (nm)** |
| --- | --- | --- | --- |
| **WT** | 2.71286 ± 0.0142308 | 2.69723 ± 0.0225867 | 2.70732 ± 0.0148137 |
| **R55V** | 2.74833 ± 0.0165458 | 2.71620 ± 0.0182589 | 2.72369 ± 0.202023 |
| **W67V** | 2.72172 ± 0.0174075 | 2.70569 ± 0.0221003 | 2.72207 ± 0.0183403 |
| **Y85I** | 2.74749 ± 0.0194050 | 2.70912 ± 0.0113079 | 2.74395 ± 0.0123868 |
| **H86S** | 2.72529 ± 0.0139794 | 2.72983 ± 0.0182114 | 2.75235 ± 0.0184122 |

**S6 Table. Replicate-averaged structural descriptors**. Combined mean ± SD values of RMSD, RMSF, and $R_{g}$ calculated across three independent 200 ns simulation runs for WT and MT IL-17A–IL-17RA complexes.

| **System** | **RMSD (mean ± SD)** | **RMSF (mean ± SD)** | **R_g_ (mean ± SD)** |
| --- | --- | --- | --- |
| **WT** | 0.2591 ± 0.0077 | 0.1456 ± 0.0099 | 2.7058 ± 0.0079 |
| **R55V** | 0.3079 ± 0.0291 | 0.1659 ± 0.0216 | 2.7294 ± 0.0168 |
| **W67V** | 0.2673 ± 0.0127 | 0.1407 ± 0.0064 | 2.7165 ± 0.0094 |
| **Y85I** | 0.2829 ± 0.0201 | 0.1476 ± 0.0105 | 2.7335 ± 0.0212 |
| **H86S** | 0.2798 ± 0.0119 | 0.1534 ± 0.0112 | 2.7358 ± 0.0145 |

**S7 Table.** **Structural network properties from single PSN analysis.** Topological parameters (Imin, linked nodes, links, hubs, hub links, communities) were evaluated for wild type and mutants (R55V, W67V, Y85I, and H86S) at the 100 ns frame.

| Parameter | Single PSN Wild | Single PSN R55V | Single PSN W67V | Single PSN Y85I | Single PSN H86S |
| --- | --- | --- | --- | --- | --- |
| Imin | 3.06 | 3.24 | 3.38 | 3.26 | 3.39 |
| Number of Linked Nodes | 489 | 495 | 497 | 499 | 491 |
| Number of Links | 575 | 578 | 574 | 583 | 571 |
| Number of Hubs | 83 | 78 | 71 | 77 | 72 |
| Number of Links Mediated by Hubs | 298 | 286 | 260 | 277 | 283 |
| Number of Communities | 23 | 28 | 24 | 28 | 26 |
| Number of Nodes Involved in Communities | 146 | 140 | 134 | 137 | 129 |
| Number of Links Involved in Communities | 191 | 172 | 169 | 170 | 165 |

**S8 Table.** **Differential network connectivity indices.** Comparison of unique versus shared network nodes, links, and hubs between the Wild-Type complex and individual point mutants.

| Parameter | Wild (R55V) | Wild+R55V (Mutated) | Wild (W67V) | Wild+W67V (Mutated) | Wild (Y85I) | Wild+Y85I (Mutated) | Wild (H86S) | Wild+H86S (Mutated) |
| --- | --- | --- | --- | --- | --- | --- | --- | --- |
| Imin | 3.06 | 3.24 | 3.06 | 3.38 | 3.06 | 3.26 | 3.06 | 3.39 |
| Linked Nodes | 489 | 495 | 489 | 497 | 489 | 499 | 489 | 491 |
| Specific Nodes (%) | 15 (3.07%) | 21 (4.24%) | 12 (2.45%) | 20 (4.02%) | 9 (1.84%) | 19 (3.81%) | 15 (3.07%) | 17 (3.46%) |
| Shared Nodes (%) | 474 (96.93%) | 474 (95.76%) | 477 (97.55%) | 477 (95.98%) | 480 (98.16%) | 480 (96.19%) | 474 (96.93%) | 474 (96.54%) |
| Number of Links | 575 | 578 | 575 | 574 | 575 | 583 | 575 | 571 |
| Specific Links (%) | 241 (41.91%) | 244 (42.21%) | 231 (40.17%) | 230 (40.07%) | 231 (40.17%) | 239 (40.99%) | 254 (44.17%) | 250 (43.78%) |
| Shared Links (%) | 334 (58.09%) | 334 (57.79%) | 344 (59.83%) | 344 (59.93%) | 344 (59.83%) | 344 (59.01%) | 321 (55.83%) | 321 (56.22%) |
| Number of Hubs | 83 | 78 | 83 | 71 | 83 | 77 | 83 | 72 |
| Specific Hubs (%) | 49 (59.04%) | 44 (56.41%) | 48 (57.83%) | 36 (50.70%) | 47 (56.63%) | 41 (53.25%) | 40 (48.19%) | 29 (40.28%) |
| Shared Hubs (%) | 34 (40.96%) | 34 (43.59%) | 35 (42.17%) | 35 (49.30%) | 36 (43.37%) | 36 (46.75%) | 43 (51.81%) | 43 (59.72%) |

**S9 Table.** **Consensus network parameters for combined chains.** Comparing network properties of wild type combined with R55V and W67V versus wild type combined with Y85I and H86S, highlighting connectivity, hubs, and community structures.

| Parameter | Consensus Wild + R55V + W67V  (Chain B Residues) | Consensus Wild + Y85I + H86S  (Chain A Residues) |
| --- | --- | --- |
| Imin | 3.22667 | 3.23667 |
| Number of Linked Nodes | 516 | 514 |
| Number of Links | 610 | 606 |
| Number of Hubs | 85 | 82 |
| Number of Links Mediated by Hubs | 311 | 310 |
| Number of Communities | 25 | 21 |
| Number of Nodes Involved in Communities | 143 | 136 |
| Number of Links Involved in Communities | 184 | 180 |

**S10 Table.** **Snapshot-wise decomposition of MM/GBSA binding free energies.** Individual energetic components for representative frames at 0, 50, 100, 150, and 200 ns for WT and mutant complexes at the Chain B with C and Chain A with C interfaces.

| **System** | **Time (ns)** | **∆E_VDW_** | **∆E_ELE_** | **∆G_GB_** | **∆G_SA_** | **∆G_MMGBSA_** |
| --- | --- | --- | --- | --- | --- | --- |
| **WT**  **(Chain B with C)** | 0 | -126.62 | -525.50 | 555.38 | -17.57 | -114.31 |
|  | 50 | -119.01 | -465.9 | 504.68 | -16.37 | -96.59 |
|  | 100 | -126.67 | -526.21 | 556.31 | -17.61 | -114.18 |
|  | 150 | -122.51 | -478.27 | 508.25 | -17.31 | -109.85 |
|  | 200 | -141.04 | -319.51 | 381.82 | -18.68 | -97.41 |
| **R55V** | 0 | -120.18 | -339.52 | 373.09 | -16.87 | -103.49 |
|  | 50 | -119.57 | -356.60 | 394.62 | -16.33 | -97.88 |
|  | 100 | -118.18 | -332.20 | 368.53 | -16.51 | -98.36 |
|  | 150 | -106.62 | -350.85 | 373.02 | -14.80 | -99.26 |
|  | 200 | -140.50 | -190.58 | 260.36 | -18.41 | -89.13 |
| **W67V** | 0 | -97.61 | -362.77 | 411.61 | -13.34 | -62.11 |
|  | 50 | -109.51 | -446.49 | 483.70 | -16.11 | -88.40 |
|  | 100 | -97.75 | -362.54 | 411.54 | -13.35 | -62.08 |
|  | 150 | -133.60 | -547.57 | 593.70 | -18.36 | -105.82 |
|  | 200 | -122.19 | -178.93 | 231.99 | -15.87 | -85.01 |
| **WT**  **(Chain A with C)** | 0 | -124.59 | -108.39 | 155.57 | -15.18 | -92.58 |
|  | 50 | -119.68 | -217.55 | 243.52 | -16.85 | -110.56 |
|  | 100 | -124.59 | -108.39 | 155.57 | -15.18 | -92.58 |
|  | 150 | -113.67 | -60.81 | 109.67 | -15.39 | -80.20 |
|  | 200 | -113.76 | -172.79 | 215.12 | -15.42 | -86.84 |
| **Y85I** | 0 | -114.87 | -181.75 | 230.93 | -14.97 | -80.66 |
|  | 50 | -119.63 | -59.65 | 104.90 | -14.17 | -88.56 |
|  | 100 | -116.65 | -206.22 | 253.44 | -15.43 | -84.85 |
|  | 150 | -100.72 | -188.49 | 228.82 | -13.07 | -73.46 |
|  | 200 | -115.50 | -192.74 | 240.77 | -15.14 | -82.60 |
| **H86S** | 0 | -96.28 | -120.07 | 158.57 | -12.87 | -70.66 |
|  | 50 | -127.24 | -85.79 | 137.11 | -14.89 | -90.81 |
|  | 100 | -113.80 | -188.85 | 237.21 | -14.89 | -80.33 |
|  | 150 | -117.46 | -71.59 | 120.94 | -14.67 | -82.78 |
|  | 200 | -115.37 | -178.84 | 229.53 | -14.83 | -79.51 |

**S11 Table.** **Per-residue binding free energies of the interfacial residues.** Per-residue energetic decomposition (in kcal/mol) of target interfacial residues in the D1 domain (R55V, W67V) and D2 domain (Y85I, H86S) of IL-17A in complex with IL-17RA.

| D1 domain Residue | Wild-type (WT) | R55V | W67V | D2 domain Residue | Wild-type (WT) | Y85I | H86S |
| --- | --- | --- | --- | --- | --- | --- | --- |
| ASN-23 | 0.17 | 0.22 | 0.28 | **ASN-89** | 0.22 | 0.32 | 0.33 |
| SER-24 | 0.19 | 0.31 | 0.24 | **ARG-93** | -2.74 | 0.23 | 0.46 |
| LEU-26 | -2.32 | -1.26 | -1.77 | **GLN-94** | -3.48 | -2.81 | -0.25 |
| CYS-26 | -1.59 | -1.00 | -0.78 | **TYR-85** | -7.59 | -2.77 | -5.90 |
| ARG-55 | -5.75 | -0.08 | -4.91 | **ASN-88** | -5.35 | -4.42 | -4.66 |
| LEU-88 | -1.76 | -0.76 | -0.84 | **ILE-127** | -4.62 | -4.52 | -4.01 |
| PRO-103 | -1.07 | -0.03 | 0.05 | **HIE-129** | -5.44 | -2.71 | -4.75 |
| PHE-110 | -2.74 | -2.35 | -2.27 | **HIE-130** | -3.74 | -2.28 | 0.12 |
| TYR-126 | -0.25 | -0.11 | -0.06 | **VAL-131** | -5.65 | -5.10 | -0.30 |
| PRO-136 | -3.46 | -0.46 | 0.09 | **LEU-202** | -2.04 | -1.94 | -1.06 |
| ILE-137 | -0.22 | 0.09 | -0.06 | **THR-204** | -0.05 | 0.06 | 0.07 |
| PRO-138 | -4.73 | -3.60 | -1.48 | **MET-218** | -2.55 | -1.13 | -2.24 |
| ASN-148 | 0.08 | 0.23 | 0.24 | **ASN-261** | -3.76 | -2.94 | -3.15 |
| CYS-165 | -0.17 | -0.08 | -0.05 | **LEU-264** | -4.45 | -3.67 | -4.03 |
| SER-167 | -0.44 | 0.13 | 0.06 | **ARG-265** | -8.24 | -7.02 | -5.81 |
| GLU-216 | 0.27 | 0.42 | 0.48 | **SER-267** | -5.44 | -1.75 | -0.22 |
