# Supplementary material for: Mutation-induced structural and dynamic perturbations at the IL-17A–IL-17RA interface: Therapeutic insights from multi-replicate molecular dynamics simulations
Source: PLoS One. 2026 Nov 6;21(11):e0361876. doi: 10.1371/journal.pone.0361876 (PMC13742950; doi:10.1371/journal.pone.0361876)
Supplement: Peer review history

# Peer review: Decision Letter 0

**DOI:** 10.1371/journal.pone.0361876.r001

**Authors:** Anirudh Shanbhag

2 Sep 2026

Dear Dr. Ul-Haq,

Thank you for submitting your manuscript to PLOS One. After careful consideration, we feel that it has merit but does not fully meet PLOS One’s publication criteria as it currently stands. Therefore, we invite you to submit a revised version of the manuscript that addresses the points raised during the review process.

Please submit your revised manuscript by Nov 01 2026 11:59PM. If you will need more time than this to complete your revisions, please reply to this message or contact the journal office at plosone@plos.org. When you're ready to submit your revision, log on to https://www.editorialmanager.com/pone/ and select the 'Submissions Needing Revision' folder to locate your manuscript file.

- A letter that responds to each point raised by the academic editor and reviewer(s). You should upload this letter as a separate file labeled 'Response to Reviewers'.
- A marked-up copy of your manuscript that highlights changes made to the original version. You should upload this as a separate file labeled 'Revised Manuscript with Track Changes'.
- An unmarked version of your revised paper without tracked changes. You should upload this as a separate file labeled 'Manuscript'.

If you would like to make changes to your financial disclosure, please include your updated statement in your cover letter. Guidelines for resubmitting your figure files are available below the reviewer comments at the end of this letter.

If applicable, we recommend that you deposit your laboratory protocols in protocols.io to enhance the reproducibility of your results. Protocols.io assigns your protocol its own identifier (DOI) so that it can be cited independently in the future. For instructions see: https://journals.plos.org/plosone/s/submission-guidelines#loc-laboratory-protocols. Additionally, PLOS One offers an option for publishing peer-reviewed Lab Protocol articles, which describe protocols hosted on protocols.io. Read more information on sharing protocols at https://plos.org/protocols?utm_medium=editorial-email&utm_source=authorletters&utm_campaign=protocols.

As the corresponding author, your ORCID iD is verified in the submission system and will appear in the published article. PLOS supports the use of ORCID, and we encourage all coauthors to register for an ORCID iD and use it as well. Please encourage your coauthors to verify their ORCID iD within the submission system before final acceptance, as unverified ORCID iDs will not appear in the published article. *Only*  the individual author can complete the verification step; PLOS staff *cannot*  verify ORCID iDs on behalf of authors.

We look forward to receiving your revised manuscript.

Kind regards,

Anirudh Shanbhag

Academic Editor

PLOS One

Journal Requirements:

When submitting your revision, we need you to address these additional requirements.

1. Please ensure that your manuscript meets PLOS ONE's style requirements, including those for file naming. The PLOS ONE style templates can be found at

https://journals.plos.org/plosone/s/file?id=wjVg/PLOSOne_formatting_sample_main_body.pdf and

https://journals.plos.org/plosone/s/file?id=ba62/PLOSOne_formatting_sample_title_authors_affiliations.pdf

2. Please note that PLOS One has specific guidelines on code sharing for submissions in which author-generated code underpins the findings in the manuscript. In these cases, we expect all author-generated code to be made available without restrictions upon publication of the work. Please review our guidelines at https://journals.plos.org/plosone/s/materials-and-software-sharing#loc-sharing-code and ensure that your code is shared in a way that follows best practice and facilitates reproducibility and reuse.

3. Thank you for stating the following financial disclosure: [Ongoing Research Funding program (ORF-2026-994), King Saud University, Riyadh, Saudi Arabia].

Please state what role the funders took in the study.  If the funders had no role, please state: ""The funders had no role in study design, data collection and analysis, decision to publish, or preparation of the manuscript.""

If this statement is not correct you must amend it as needed.

Please include this amended Role of Funder statement in your cover letter; we will change the online submission form on your behalf.

4. Thank you for stating the following in the Acknowledgments Section of your manuscript: [The authors extend their appreciation to the Ongoing Research Funding program (ORF-2026-994), King Saud University, Riyadh, Saudi Arabia.]

We note that you have provided funding information that is not currently declared in your Funding Statement. However, funding information should not appear in the Acknowledgments section or other areas of your manuscript. We will only publish funding information present in the Funding Statement section of the online submission form.

Please remove any funding-related text from the manuscript and let us know how you would like to update your Funding Statement. Currently, your Funding Statement reads as follows: [Ongoing Research Funding program (ORF-2026-994), King Saud University, Riyadh, Saudi Arabia].

Please include your amended statements within your cover letter; we will change the online submission form on your behalf.

5. We note that Figures 1,9,10,11, and Fig S12, Fig S13 in your submission contain copyrighted images. All PLOS content is published under the Creative Commons Attribution License (CC BY 4.0), which means that the manuscript, images, and Supporting Information files will be freely available online, and any third party is permitted to access, download, copy, distribute, and use these materials in any way, even commercially, with proper attribution. For more information, see our copyright guidelines: http://journals.plos.org/plosone/s/licenses-and-copyright.

We require you to either (1) present written permission from the copyright holder to publish these figures specifically under the CC BY 4.0 license, or (2) remove the figures from your submission:

1. You may seek permission from the original copyright holder of Figures 1,9,10,11, and Fig S12, Fig S13 to publish the content specifically under the CC BY 4.0 license.

We recommend that you contact the original copyright holder with the Content Permission Form (http://journals.plos.org/plosone/s/file?id=7c09/content-permission-form.pdf) and the following text:

“I request permission for the open-access journal PLOS ONE to publish XXX under the Creative Commons Attribution License (CCAL) CC BY 4.0 (http://creativecommons.org/licenses/by/4.0/). Please be aware that this license allows unrestricted use and distribution, even commercially, by third parties. Please reply and provide explicit written permission to publish XXX under a CC BY license and complete the attached form.”

Please upload the completed Content Permission Form or other proof of granted permissions as an "Other" file with your submission.

In the figure caption of the copyrighted figure, please include the following text: “Reprinted from [ref] under a CC BY license, with permission from [name of publisher], original copyright [original copyright year].”

2. If you are unable to obtain permission from the original copyright holder to publish these figures under the CC BY 4.0 license or if the copyright holder’s requirements are incompatible with the CC BY 4.0 license, please either i) remove the figure or ii) supply a replacement figure that complies with the CC BY 4.0 license. Please check copyright information on all replacement figures and update the figure caption with source information. If applicable, please specify in the figure caption text when a figure is similar but not identical to the original image and is therefore for illustrative purposes only.

6. Please include captions for your Supporting Information files at the end of your manuscript, and update any in-text citations to match accordingly. Please see our Supporting Information guidelines for more information: http://journals.plos.org/plosone/s/supporting-information.

7. If the reviewer comments include a recommendation to cite specific previously published works, please review and evaluate these publications to determine whether they are relevant and should be cited. There is no requirement to cite these works unless the editor has indicated otherwise.

[Note: HTML markup is below. Please do not edit.]

Reviewers' comments:

Reviewer's Responses to Questions

**Comments to the Author**

1. Is the manuscript technically sound, and do the data support the conclusions?

Reviewer #1: Partly

Reviewer #2: Yes

**********

2. Has the statistical analysis been performed appropriately and rigorously?

Reviewer #1: N/A

Reviewer #2: Yes

**********

3. Have the authors made all data underlying the findings in their manuscript fully available?

The PLOS Data policy

Reviewer #1: No

Reviewer #2: Yes

**********

4. Is the manuscript presented in an intelligible fashion and written in standard English?

Reviewer #1: Yes

Reviewer #2: Yes

**********

Reviewer #1: The study is based on the IL-17A/IL-17RA crystallographic structure (PDB 4HSA) and introduces, in silico, four mutations that replace IL-17A residues with their IL-17F equivalents. Utilising molecular dynamics simulations in triplicate, in conjunction with PSN, PCA/FEL, DCCM, and MM/GBSA methodologies, the study posits that the four mutations engender the destabilisation of the complex and diminish its binding affinity. The initial system and the mutations have prior experimental support, which lends credibility to the work. Nevertheless, the study has some areas for improvement, which will be discussed below.

• The most significant issue pertains to the binding energies. The MM/GBSA calculation is based on a single final frame, without ensemble averaging, without entropy, and using the ff02 force field, which differs from the ff99SB-ILDN force field used in the dynamics simulations. The text itself acknowledges that HawkDock does not provide an error estimate. The fragility is self-evident: In the transition from the initial to the final frame, W67V undergoes a significant shift in its energy level, with a notable decrease from −62.11 to −85.01 kcal/mol. It is imperative to acknowledge that a central quantitative assertion cannot be substantiated by a solitary number in the absence of sampling or uncertainty.

• Secondly, the absence of statistical data throughout the manuscript is problematic. The execution of three replicates is undertaken for the sole purpose of confirming convergence. The differences that have been purported to be of significance are negligible in size or commensurate with their own standard deviation. The root-mean-square deviation (RMSD) of the wild-type sequence in comparison with Y85I is found to be entirely congruent. The Rg undergoes a 1.3% alteration, which is characterised as "unfolding". It is imperative to note that, in the absence of a comparison between replicates, it becomes unfeasible to distinguish the signal from thermal noise. The term 'significant' should be reserved for findings that have been substantiated by statistical testing.

• Thirdly, an inherent internal contradiction is evident. The conclusion of the study indicates that mutants exhibit a reduced degree of flexibility in comparison to the wild-type, a finding that stands in stark contrast to the overall body of results, which suggest the converse. The direction of the effect remains unknown to the reader. The conclusion must be rewritten in accordance with the data.

• Fourthly, a hydrogen bond occupancy of 117.16% is reported for GLU57, and 34 bonds are attributed to a single residue. It is imperative to note that the maximum permissible occupancy is restricted to 100%. This finding suggests the presence of either a non-standard definition or an error in the calculation of a section that provides support for the mechanistic argument.

• In the fifth instance, the figure caption for Figure 7 is not consistent with the order of Chains A and B as presented in the rest of the text. The RMSF has identified the W67V and Y85I mutations in "residues 430–500 of Chain C", which correspond to the receptor. It is evident that Section 3.8 has assigned a specific designation to the values from Chain B, designating them as "Chain A". It is imperative that the A/B/C nomenclature is standardised, and that the figure captions are corrected.

Reviewer #2: The interaction between Interleukin-17A (IL-17A) and its receptor IL-17RA is regarded as an important of drug design toward treatment of inflammatory autoimmune diseases. The authors applied molecular dynamics simulations, MM-GBSA calculations, principal component analysis and free energy landscapes to inhibiting mechanism on interactions of IL-17A with IL-17RA via inhibitors. The results revealed how mutations affected the structure, contact networks, dynamics, and binding affinity of the IL-17A/IL-17RA complex. Overall, this is an interesting study. Recommending that their work can be accepted after minor revision.

1. In their equation 1, the “g” had better be revised as “G”.

2. The authors had better revise the description on “Parameter trajectories”.

3. The standard errors should be added to their Figure 2 and 3 so that the reliability of their results can be accessed.

4. In their free energy landscapes, the authors had better use the same free energy scale in their color bar.

5. In their introduction, the authors mentioned the significance of multiple replica MD simulation in their study. Two works relating with multiple replica simulations (DOI: 10.1016/j.ijbiomac.2026.151977 and 10.1021/acs.jpcb.5c04050) should be mentioned.

6. The authors calculated binding free energies and residue contributions to binding. The authors should simply discuss how their results are used to guide future drug design.

**********

what does this mean?). If published, this will include your full peer review and any attached files.

If you choose “no”, your identity will remain anonymous but your review may still be made public.

**Do you want your identity to be public for this peer review?**  For information about this choice, including consent withdrawal, please see our Privacy Policy

Reviewer #1: No

Reviewer #2: No

**********

[NOTE: If reviewer comments were submitted as an attachment file, they will be attached to this email and accessible via the submission site. Please log into your account, locate the manuscript record, and check for the action link "View Attachments". If this link does not appear, there are no attachment files.]

To ensure your figures meet our technical requirements, please review our figure guidelines: https://journals.plos.org/plosone/s/figures

You may also use PLOS’s free figure tool, NAAS, to help you prepare publication quality figures: https://journals.plos.org/plosone/s/figures#loc-tools-for-figure-preparation.

NAAS will assess whether your figures meet our technical requirements by comparing each figure against our figure specifications.

# Author comment: Author response to Decision Letter 1

**DOI:** 10.1371/journal.pone.0361876.r002

2 Oct 2026

Response to Reviewers

Response Report

Article: "Mutation-Induced Structural and Dynamic Perturbations at the IL-17A–IL-17RA Interface: Therapeutic Insights from Multi-Replicate Molecular Dynamics Simulations"

Reviewer 1’s Comments

The study is based on the IL-17A/IL-17RA crystallographic structure (PDB 4HSA) and introduces, in silico, four mutations that replace IL -17A residues with their IL -17F equivalents. Utilising molecular dynamics simulations in triplicate, in conjunction with PSN, PCA/FEL, DCCM, and MM/GBSA methodologies, the study posits that the four mutations engender the destabilisation of the complex and diminish its binding affinity. The initial system and the mutations have prior experimental support, which lends credibility to the work. Nevertheless, the study has some areas for improvement, which will be discussed below.

Comment 1: The most significant issue pertains to the binding energies. The MM/GBSA calculation is based on a single final frame, without ensemble averaging, without entropy, and using the ff02 force field, which differs from the ff99SB -ILDN force field used in the dynamics simulations. The text itself acknowledges that HawkDock does not provide an error estimate. The fragility is self -evident: In the transition from the initial to the final frame, W67V undergoes a significant shift in its energy level, with a notable decrease from −62.11 to −85.01 kcal/mol. It is imperative to acknowledge that a central quantitative assertion cannot be substantiated by a solitary number in the absence of sampling or uncertainty.

Author Response: Thank you for this valuable comment. We agree that a single final-frame MM/GBSA calculation does not adequately capture conformational variability during MD simulations. To address this concern, we recalculated the MM/GBSA energies using five snapshots extracted at 0, 50, 100, 150, and 200 ns from the primary trajectory, tracing the structural relaxation from the un-equilibrated initial complex to the equilibrated state. For each representative structure, the MM/GBSA energy component results are reported as mean ± standard deviation, providing an estimate of variability across the sampled conformations. The revised results are presented in Table 2, while the complete snapshot-wise calculations are provided in Supplementary S10 Table. Methods Section 2.8 and Results Section 3.8 have also been revised accordingly. In addition, the limitations of the HawkDock implementation, including the absence of an explicit configurational entropy contribution, have been acknowledged in the revised Methodology section. Similar workflows using different force fields for MD simulations and subsequent HawkDock MM/GBSA calculations (CHARMM36m/ff02, OPLS3e/ff02, and GROMOS96 54a7/ff02) have also been reported previously (doi:10.3390/v14010001; doi:10.1021/acsomega.3c09253; doi:10.1039/D2CP03012H). Based on the expanded ensemble analys is, W67V exhibited an average binding free energy of −80.68 ± 18.72 kcal/mol, compared with −106.47 ± 8.83 kcal/mol for WT, indicating markedly weaker predicted binding overall for W67V. These revisions provide a more representative and transparent assessment of mutation -induced changes in binding energetics.

Comment 2: Secondly, the absence of statistical data throughout the manuscript is problematic. The execution of three replicates is undertaken for the sole purpose of confirming convergence. The differences that have been purported to be of significance are negligibl e in size or commensurate with their own standard deviation. The root-mean-square deviation (RMSD) of the wild-type sequence in comparison with Y85I is found to be entirely congruent. The Rg undergoes a 1.3% alteration, which is characterised as "unfolding". It is imperative to note that, in the absence of a comparison between replicates, it becomes unfeasible to distinguish the signal from thermal noise. The term 'significant' should be reserved for findings that have been substantiated by statistical testing.

Author Response : We sincerely thank the reviewer for this important critique regarding trajectory sampling, replica usage, and terminology. We agree that comparison of the independent MD replicas is important for evaluating the reproducibility and variability of the observed conformational behavior. Accordingly, the MD trajectory analyses were re-evaluated across three independent replicas for all five systems. The RMSD, RMSF, and Rg analyses were subsequently summarized as comparative bar graphs with SD error bars in the main manuscript (Figs 5, 7, and 9, respectively), providing a direct comparison of the variability observed among replicas. The corresponding numerical values and detailed replica -wise results are provided in Supplementary S3-S6 Tables, while the individual plots for Replicates 2 and 3 are provided in Supplementary S3-S11 Figs. We have also revised the manuscript to remove unsupported use of terms and to interpret the observed differences descriptively in the context of inter -replica variability. These revisions provide a more quantitative and appropriately cautious assessment of the results and minimize the possibility of interpreting normal simulation variability as meaningful structural effects.

Comment 3: Thirdly, an inherent internal contradiction is evident. The conclusion of the study indicates that mutants exhibit a reduced degree of flexibility in comparison to the wild -type, a finding that stands in stark contrast to the overall body of results, which suggest the converse. The direction of the effect remains unknown to the reader. The conclusion must be rewritten in accordance with the data.

Author Response : Thank you for highlighting this important inconsistency. Accordingly, we have thoroughly revised the Conclusion, which now describes the observed effects as localized changes in structural flexibility rather than assigning a single overall direction to the flexibility changes. These revisions resolve the inconsistency and ensure that the Conclusion is fully aligned with the corresponding results presented in the manuscript.

Comment 4: Fourthly, a hydrogen bond occupancy of 117.16% is reported for GLU57, and 34 bonds are attributed to a single residue. It is imperative to note that the maximum permissible occupancy is restricted to 100%. This finding suggests the presence of either a non -standard definition or an error in the calculation of a section that provides support for the mechanistic argument.

Author Response: We sincerely thank the reviewer for highlighting this point and allowing us to clarify the hydrogen-bond calculation using the VMD HBonds plugin. The reported value of 117.16% for GLU57 represents cumulative residue -level hydrogen-bond occupancy, rather than the occupancy of a single hydrogen bond. Hydrogen bonds were analyzed using the VMD built-in H-Bonds plugin with a donor–acceptor distance cutoff of 3.5 Å. GLU57 contains two charged side-chain carboxylate oxygen acceptors (OE1 and OE2), capable of forming bifurcated (multidentate) hydrogen bonds with multiple donor atoms . VMD reports the occupancy of each distinct donor -acceptor pair separately, and the occupancies of multiple pairs involving GLU57 were summed to obtain the cumulative residue-level value, which can exceed 100% when multiple hydrogen-bond interactions contribute to the same residue. Similarly, the reported 34 hydrogen bonds represent 34 distinct donor-acceptor atom-pair combinations detected during the trajectory, rather than 34 simultaneously formed hydrogen bonds. To ensure clarity, we have revised the Methodology Section 2.4 , Results and Discussion Section 3.3 , and Fig 11 caption to explicitly define cumulative residue-level occupancy and distinguish it from individual hydrogen-bond occupancy.

Comment 5: In the fifth instance, the figure caption for Figure 7 is not consistent with the order of Chains A and B as presented in the rest of the text. The RMSF has identified the W67V and Y85I mutations in "residues 430–500 of Chain C", which correspond to the receptor. It is evident that Section 3.8 has assigned a specific designation to the values from Chain B, designating them as "Chain A". It is imperative that the A/B/C nomenclature is standardised, and that the figure captions are corrected.

Author Response: We thank the reviewer for identifying this inconsistency. We agree that the original wording and figure caption could lead to ambiguity regarding the chain assignments. Accordingly, we have corrected the chain nomenclature in the Fig 10 caption (revised manuscript) and revised the RMSF description to clearly indicate that the increased fluctuations were observed in residues 430 –500 of the receptor (Chain C) in the W67V and Y85I mutant complexes, rather than implying that these mutations are located within Chain C. In addition, we carefully reviewed the manuscript and standardized the chain A/B/C nomenclature throughout the text and figure captions to ensure consistency with the original IL-17A/IL-17RA structure used in this study.

Reviewer 2’s Comments

The interaction between Interleukin -17A (IL -17A) and its receptor IL -17RA is regarded as an important of drug design toward treatment of inflammatory autoimmune diseases. The authors applied molecular dynamics simulations, MM -GBSA calculations, principal component analysis and free energy landscapes to inhibiting mechanism on interactions of IL -17A with IL-17RA via inhibitors. The results revealed how mutations affected the structure, contact networks, dynamics, and binding affinity of the IL -17A/IL-17RA comple x. Overall, this is an interesting study. Recommending that their work can be accepted after minor revision.

Comment 1: In their equation 1, the “g” had better be revised as “G”.

Author Response : We thank the reviewer for identifying this typographical error. We have revised Equation (1) to use "G" instead of "g" as suggested, to ensure consistency and accuracy throughout the manuscript.

Comment 2: The authors had better revise the description on “Parameter trajectories”.

Author Response: We thank the reviewer for this valuable suggestion. We have revised the concluding paragraph of Section 2.4 to provide a clearer and more rigorous description of the parameter trajectories, including the analyzed molecular dynamics metrics and their role in assessing structural stability, flexibility, and intermolecular interactions.

Comment 3: The standard errors should be added to their Figure 2 and 3 so that the reliability of their results can be accessed.

Author Response: We thank the reviewer for the suggestion. We clarify that Figures 2 and 3 report deterministic, single -structure mutagenesis predictions from web -based algorithms, not replicate measurements. Each algorithm returns a single fixed value per structure/mutation, so no distribution exists from which a standard error could be calculated. This screening step was necessary because prior experimental work did not specify which chain of the IL-17A homodimer carried the mutation. Since the two chains are structurally equivalent, we evaluated candidate mutations on both chains, identifying R55V and W67V (Chain B) and Y85I and H86S (Chain A) as having notable predicted effects. These four candidates were selected for multi -replicate MD simulations, with ensemble metrics reported as the mean ± SD and detailed replicate breakdowns are provided in Supplementary Information . To address the reviewer’s concern and avoid potential ambiguity regarding the interpretation of these results, we have revised the manuscript to clearly describe the deterministic nature of the computational mutagenesis predictions and their specific role as an initial screening assessment prior to MD simulations. Appropriate clarifications have been incorporated into Section 3.1 and the figure caption.

Comment 4: In their free energy landscapes, the authors had better use the same free energy scale in their color bar.

Author Response: We sincerely thank the reviewer for this suggestion. We would like to clarify that the free -energy landscapes were calculated independently for each complex from its respective MD trajectory. Therefore, the free -energy values are relative to the most populated conformational state within each system. Thus, each complex has its own actual free -energy distribution and numerical range, reflecting its distinct PC1/PC2 conformational sampling. These ranges were retained as calculated and were not artificially adjusted for visualization. Applying a single fixed scale could compress the energy distribution of systems with narrower ranges and obscure their low-energy basins. To make this point explicit and avoid ambiguity for readers, we have clarified in the revised manuscript and Fig 15 caption that the color bars represent the actual relative free-energy range calculated for each individual complex and that the values are referenced to the corresponding system's most populated state.

Comment 5: In their introduction, the authors mentioned the significance of multiple replica MD simulation in their study. Two works relating with multiple replica simulations (DOI: 10.1016/j.ijbiomac.2026.151977 and 10.1021/acs.jpcb.5c04050) should be mentioned.

Author Response : We thank the reviewer for this valuable suggestion. We agree that highlighting recent studies on multiple -replica MD simulations strengthens the rationale of our work. Accordingly, we have revised the final paragraph of the Introduction to emphasize the importance of replica simulations in overcoming energy barriers, reducing kinetic trapping, and improving the robustness of conformational sampling and statistical convergence. The two recommended studies have also been incorporated as References 37 and 38.

Comment 6: The authors calculated binding free energies and residue contributions to binding. The authors should simply discuss how their results are used to guide future drug design.

Author Response : We thank the reviewer for this valuable suggestion. We agree that

connecting the MM-GBSA binding free energies and per -residue decomposition results to future drug design enhances the practical significance of our findings. Accordingly, we have added a paragraph to Section 3.8 discussing how the identified interface hotspots may guide future structure-based ligand optimization and IL-17A inhibitor development.

Attachment Submitted filename: Response-to-comments.docx

# Peer review: Decision Letter 1

**DOI:** 10.1371/journal.pone.0361876.r003

**Authors:** Anirudh Shanbhag

19 Oct 2026

Mutation-Induced Structural and Dynamic Perturbations at the IL-17A–IL-17RA Interface: Therapeutic Insights from Multi-Replicate Molecular Dynamics Simulations

PONE-D-26-39786R1

Dear Dr. Ul-Haq,

We’re pleased to inform you that your manuscript has been judged scientifically suitable for publication and will be formally accepted for publication once it meets all outstanding technical requirements.

Within one week, you’ll receive an e-mail detailing the required amendments. When these have been addressed, you’ll receive a formal acceptance letter and your manuscript will be scheduled for publication.

An invoice will be generated when your article is formally accepted. Please note, if your institution has a publishing partnership with PLOS and your article meets the relevant criteria, all or part of your publication costs will be covered. Please make sure your user information is up-to-date by logging into Editorial Manager at Editorial Manager® and clicking the ‘Update My Information' link at the top of the page. For questions related to billing, please contact billing support.

If your institution or institutions have a press office, please notify them about your upcoming paper to help maximize its impact. If they’ll be preparing press materials, please inform our press team as soon as possible -- no later than 48 hours after receiving the formal acceptance. Your manuscript will remain under strict press embargo until 2 pm Eastern Time on the date of publication. For more information, please contact onepress@plos.org.

Kind regards,

Anirudh Shanbhag

Academic Editor

PLOS One

Additional Editor Comments (optional):

Reviewers' comments:

Reviewer's Responses to Questions

**Comments to the Author**

Reviewer #1: All comments have been addressed

Reviewer #2: All comments have been addressed

**********

2. Is the manuscript technically sound, and do the data support the conclusions?

Reviewer #1: (No Response)

Reviewer #2: Yes

**********

3. Has the statistical analysis been performed appropriately and rigorously?

Reviewer #1: (No Response)

Reviewer #2: Yes

**********

4. Have the authors made all data underlying the findings in their manuscript fully available?

The PLOS Data policy

Reviewer #1: (No Response)

Reviewer #2: Yes

**********

5. Is the manuscript presented in an intelligible fashion and written in standard English?

Reviewer #1: (No Response)

Reviewer #2: Yes

**********

Reviewer #1: (No Response)

Reviewer #2: The authors have provided rational responses to my comments and their work can be accepted in its current state.

**********

what does this mean?). If published, this will include your full peer review and any attached files.

If you choose “no”, your identity will remain anonymous but your review may still be made public.

**Do you want your identity to be public for this peer review?**  For information about this choice, including consent withdrawal, please see our Privacy Policy

Reviewer #1: No

Reviewer #2: No

**********

# Editor report: Acceptance letter

**DOI:** 10.1371/journal.pone.0361876.r004

**Authors:** Anirudh Shanbhag

PONE-D-26-39786R1

PLOS One

Dear Dr. Ul-Haq,

I'm pleased to inform you that your manuscript has been deemed suitable for publication in PLOS One. Congratulations! Your manuscript is now being handed over to our production team.

At this stage, our production department will prepare your paper for publication. This includes ensuring the following:

* All references, tables, and figures are properly cited

* All relevant supporting information is included in the manuscript submission,

* There are no issues that prevent the paper from being properly typeset

You will receive further instructions from the production team, including instructions on how to review your proof when it is ready. Please keep in mind that we are working through a large volume of accepted articles, so please give us a few days to review your paper and let you know the next and final steps.

Lastly, if your institution or institutions have a press office, please let them know about your upcoming paper now to help maximize its impact. If they'll be preparing press materials, please inform our press team within the next 48 hours. Your manuscript will remain under strict press embargo until 2 pm Eastern Time on the date of publication. For more information, please contact onepress@plos.org.

You will receive an invoice from PLOS for your publication fee after your manuscript has reached the completed accept phase. If you receive an email requesting payment before acceptance or for any other service, this may be a phishing scheme. Learn how to identify phishing emails and protect your accounts at https://explore.plos.org/phishing.

If we can help with anything else, please email us at customercare@plos.org.

Thank you for submitting your work to PLOS One and supporting open access.

Kind regards,

PLOS One Editorial Office Staff

on behalf of

Dr. Anirudh Shanbhag

Academic Editor

PLOS One
